# Recent Advances in Asymmetric Organocatalyzed Conjugate Additions to Nitroalkenes

**DOI:** 10.3390/molecules22060895

**Published:** 2017-05-29

**Authors:** Diego A. Alonso, Alejandro Baeza, Rafael Chinchilla, Cecilia Gómez, Gabriela Guillena, Isidro M. Pastor, Diego J. Ramón

**Affiliations:** Department of Organic Chemistry and Institute of Organic Synthesis (ISO), Faculty of Sciences, University of Alicante, PO Box 99, 03080 Alicante, Spain

**Keywords:** nitroalkenes, Michael addition, organocatalysis, asymmetric synthesis

## Abstract

The asymmetric conjugate addition of carbon and heteroatom nucleophiles to nitroalkenes is a very interesting tool for the construction of highly functionalized synthetic building blocks. Thanks to the rapid development of asymmetric organocatalysis, significant progress has been made during the last years in achieving efficiently this process, concerning chiral organocatalysts, substrates and reaction conditions. This review surveys the advances in asymmetric organocatalytic conjugate addition reactions to α,β-unsaturated nitroalkenes developed between 2013 and early 2017.

## 1. Introduction

Asymmetric organocatalysis is still a relatively young field for the chemical research community. Thus, not too many years ago, the term ‘catalysis’ was normally associated to transition metal-mediated reactions or to enzyme-aided biocatalysis. However, small organic molecules can achieve remarkably stereoselective and efficient transformations. In addition, the employed organocatalysts are usually of low molecular weight, easy to synthesize, chemically robust, and affordable. Additionally, the organocatalytic reactions are often carried out under virtually ‘open-flask’ conditions. All these reasons have made asymmetric organocatalysis a nowadays fast-forwarding topic, which shows continuous and rapid developments [1,2,3].

Among all the array of asymmetric organocatalyzed reactions, the conjugate addition reaction of carbon nucleophiles to electron-deficient alkenes is one of the most important ways of creating C-C bonds [4,5,6,7,8,9,10,11,12]. Particularly, the asymmetric organocatalyzed conjugate addition of carbon nucleophiles to α,β-unsaturated nitroalkenes has attracted considerable attention, as the final enantioenriched γ-nitrocarbonyl compounds can be transformed into compounds of interest [13,14,15,16,17].

The present review covers asymmetric organocatalytic conjugate addition reactions to α,β-unsaturated nitroalkenes published from 2013 till the first quarter of 2017, concerning substrates, organocatalysts and reactions conditions. The review has been classified depending on the nucleophile employed in the conjugate addition. Thus, Michael reactions of carbon nucleophiles such as aldehydes, ketones, 1,3-dicarbonyl compounds, nitroalkanes and heterocycles will be presented, although these systems should be considered as ‘pro-nucleophiles’, the real nucleophiles being enamines, enolates or nitronates. Finally, recent developments in asymmetric organocatalytic enantioselective hetero-Michael reactions will also be considered.

## 2. Carbon Nucleophiles

**Scheme 1:**
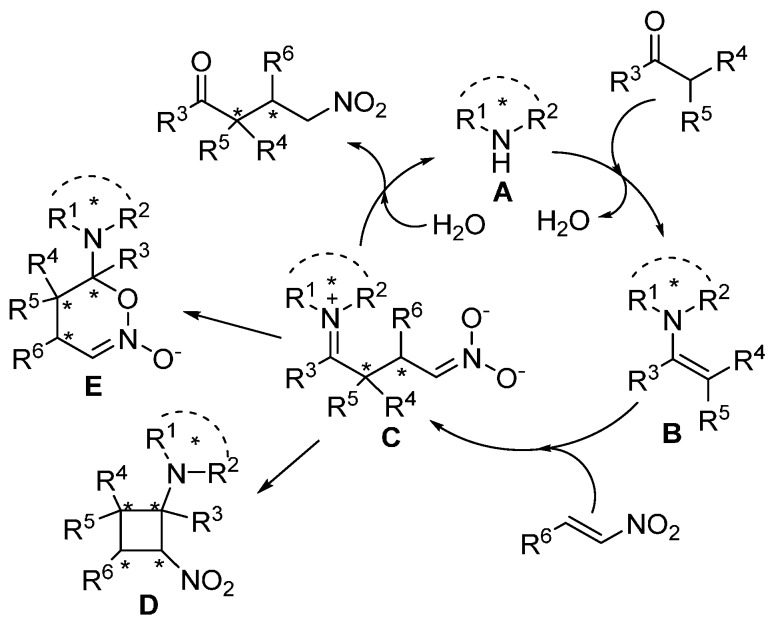
Catalytic cycle of the conjugate addition of aldehydes and ketones to nitroalkenes promoted by primary or secondary chiral amines.

Aldehydes and ketones have been the most frequently employed carbon nucleophiles (in fact, pro-nucleophiles) in the asymmetric organocatalyzed Michael addition reaction to nitroalkenes. Chiral primary and secondary amines have been used as organocatalysts, the presence of additives as co-catalysts, mainly carboxylic acids, being frequently necessary to achieve good optical and chemical yields. Concerning the mechanism of this process, although there have been suggestions that enols are the involved nucleophilic species, the nowadays accepted catalytic cycle for this reaction involves enamines as nucleophiles (Scheme 1). Thus, the chiral amine organocatalyst **A** would react with the carbonyl compound forming an enamine **B** (for primary amines, R^1^ = H, initially an imine in tautomeric equilibrium with the enamine) which would add stereoselectively to the nitroolefin, leading to the nitronate adduct **C**. This intermediate is then hydrolyzed driving to the final γ-nitrocarbonyl compound and the initial amine organocatalyst. Recent mass spectrometry studies support this mechanism [18,19]. It is interesting to remark that formation of cyclobutane **D** and 1,2-oxazine *N*-oxide **E** derivatives has been observed in this process, these compounds being resting states of the organocatalyst [20,21,22,23,24]. Its formation would ‘remove’ the amine catalyst from the cycle, which would explain the mentioned frequent necessity of adding acid co-catalysts for achieving good results. The presence of an acid not only would promote a faster imine-enamine equilibrium, but also would protonate the nitronate **C**, blocking the formation of the **D** and **E** as byproducts. This could also be achieved internally if the amine catalyst bears an acidic functionality [25]. In addition, there are evidences that, at least in some cases, support the re-formation of an enamine from intermediates **C**/**D**/**E**, with stereoselectivity now being controlled by the diastereoselectivity of enamine protonation [26]. Moreover, the presence of organic bases has sometimes shown a positive effect as co-catalyst, as they also can accelerate the reaction after favoring the creation of the enamine intermediate [27]. Furthermore, if suitable H-forming groups are also present in the chiral amine catalyst (bifunctional organocatalysts), the nitro group of the nitroalkene will be coordinated. Therefore, the enamine and the electrophile will be close enough to get a high enantioinduction.

In the case of pro-nucleophiles bearing much more acidic α-hydrogens than those on aldehydes and ketones (i.e., in the methylene group of 1,3-dicarbonyl compounds), chiral tertiary amines are usually employed as organocatalysts. Their basic character allows to deprotonate the pro-nucleophile, leading to an enolate which coordinates with the protonated base. Similarly to aldehydes and ketones, if amine-containing bifunctional organocatalysts are employed, the nitro group of the nitroalkene can be also coordinated by means of hydrogen bonds with the acidic moiety of the catalyst, affording a tight transition state which lead to suitable enantioinductions.

The present section has been classified according to the carbon pro-nucleophiles employed in the asymmetric addition to nitroalkenes. Firstly, recent reactions with enamine-forming compounds, such as aldehydes and ketones, will be presented. Secondly, more acidic systems where enolates and nitronates are involved, such as 1,3-dicarbonyl compounds and nitroalkanes, respectively, will be shown. Finally, a section dealing to the use of carbon pro-nucleophiles contained into heterocyclic systems will be considered.

### 2.1. Aldehydes

l-Proline was the first organocatalyst employed in the asymmetric conjugate addition reaction of carbonyl compounds to nitroalkenes [28,29]. Its bifunctional character, suitable to form an enamine and coordinate the nitro group of the electrophile at the same time via hydrogen bond, made it a simple and easily available organocatalyst, although poorly effective. Still nowadays, most of the organocatalysts designed for the asymmetric Michael addition of aldehydes and ketones to nitroalkenes are based in the presence of the pyrrolidine ring for the formation of the transient enamine. Their differences relay mainly in the functional group at C-2 intended to coordinate the nitro group of the nitroalkene with the highest efficiency.

**Scheme 2:**
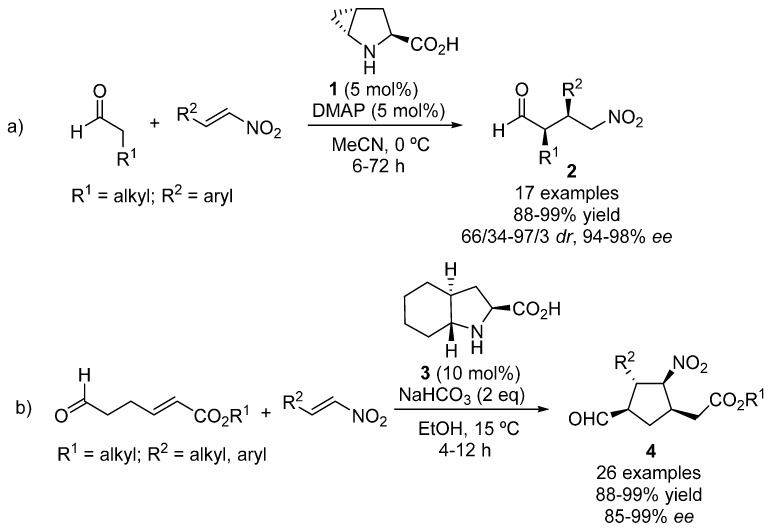
Asymmetric addition of aldehydes to nitroalkenes catalyzed by l-proline-related organocatalysts.

Closely related to the use of l-proline as organocatalyst is the recent development of rigid chiral bicyclic prolines, such as 4,5-methano-l-proline (**1**), which showed high efficiency in the Michael addition of aldehydes to β-arylated *trans*-nitroalkenes giving mainly the corresponding *syn*-nitroaldehydes **2**, the presence of *N*,*N*-4-dimethylaminopyridine (DMAP) as co-catalyst being required (Scheme 2a) [30]. Another example of proline-like organocatalyst is shown in the use of perhydroindolic acid **3** promoting the reaction between aldehydes containing an α,β-unsaturated ester and nitroolefins in the presence of sodium bicarbonate, which allows the asymmetric formation of polysubstituted aliphatic cyclopentanes **4** after a domino double Michael addition reaction (Scheme 2b) [31].

**Figure 1:**
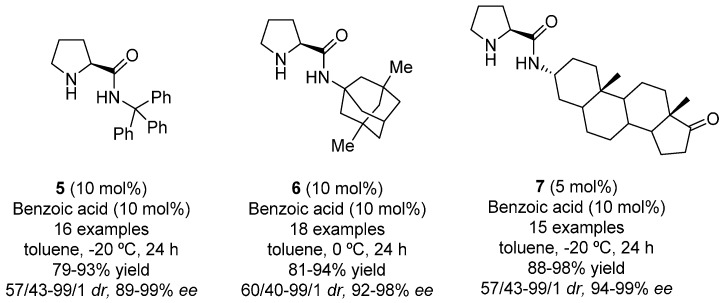
Results obtained using some l-prolinamide organocatalysts in the asymmetric addition of aldehydes to nitroalkenes leading to nitroaldehydes **2**.

Chiral organocatalysts suitable for the asymmetric Michael addition reaction of aldehydes to nitroolefins have been obtained by amidation of l-proline with different amines. The acidic hydrogen into the amide functionality allows coordination with the nitro group favoring the enantiodifferentiation in the enamine attack. Examples of these type of organocatalysts are the triphenylmethylamine-derived prolinamide **5** [32], the adamantyl l-prolinamide **6** [33] and the epiandrosterone-derived prolinamide **7** [34] (Figure 1). All of them have been used as organocatalysts in the conjugate addition of aldehydes to *trans*-nitroalkenes (using **5**: R^1^ = alkyl, R^2^ = aryl; using **6**: R^1^ = alkyl, R^2^ = alkyl, aryl; using **7**: R^1^ = alkyl, R^2^ = aryl) which leads to the *syn*-adducts **2** (Scheme 2a), the addition of benzoic acid as additive resulting crucial in all cases.

**Figure 2:**
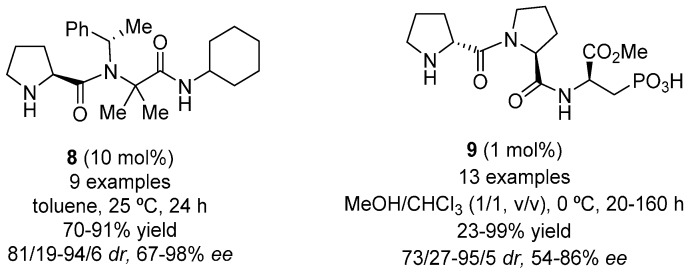
Results obtained using some peptide-derived organocatalysts in the asymmetric addition of aldehydes to nitroalkenes leading to nitroaldehydes **2**.

Peptides containing the proline moiety have also been used as organocatalysts for the asymmetric conjugate addition of aldehydes to *trans*-β-nitrostyrenes affording γ-nitroaldehydes **2** (R^1^ = alkyl, R^2^ = aryl) (Scheme 2a). Examples are the peptide-peptoid hybrid **8** [35] or the phosphonic acid-containing tripeptide **9** [36] (Figure 2). Although not too enantioselective, it is interesting to remark the low catalyst loading employed when using the tripeptide **9** and that it can be recovered by extraction due to their high solubility in water, allowing its reuse in ten reaction cycles.

**Scheme 3:**
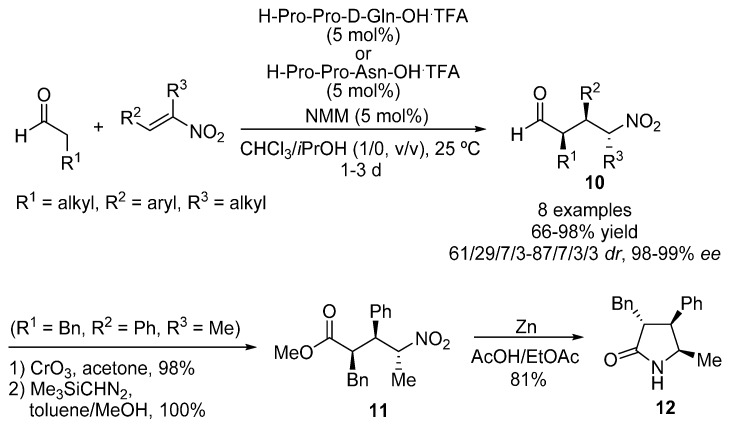
Asymmetric addition of aldehydes to α,β-disubstituted nitroalkenes catalyzed by proline-containing tripeptides and further transformation to lactams.

Another example related to proline-containing tripeptides as organocatalysts is the use of H-Pro-Pro-D-Gln-OH·TFA or H-Pro-Pro-Asn-OH·TFA (TFA = trifluoroacetic acid), combined to *N*-methylmorpholine (NMM), in the asymmetric Michael addition of aldehydes to α,β-disubstituted nitroolefins, affording γ-nitroaldehydes **10** bearing three consecutive stereogenic centers (Scheme 3) [37]. The synthetic usefulness of these nitroaldehydes is shown in the formation of fully substituted β-lactam **12**, obtained from **10** after oxidation of the aldehyde and esterification to give nitroester **11** followed by reduction of the nitro group to the amine and subsequent cyclization. The authors have also used an immobilized tripeptide as organocatalyst for the asymmetric addition of aldehydes to β-substituted nitroolefins, which results suitable to be reused 30 times and employed in a continuous flow system [37].

**Scheme 4:**
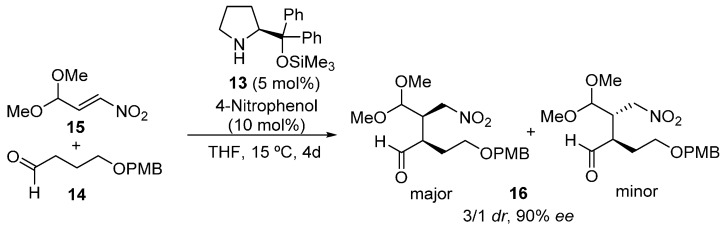
Synthesis of precursor of isoprostanes using organocatalyst **13**.

The diarylated prolinol silyl ether Hayashi-Jørgensen organocatalyst **13** has been successfully employed in the asymmetric conjugate addition of carbonyl compounds to nitroolefins [38,39]. Recently, this organocatalyst **13** has also been used in the conjugate addition of *p*-methoxybenzylated (PMB) aldehyde **14** to ketalyzed nitroalkene **15**, giving nitroaldehyde **16** as a 3/1 mixture of diastereomers employed in the total synthesis of the isoprostane isoketal 5-D_2_-IsoK, a prostaglandin-like product (Scheme 4) [40].

**Scheme 5:**
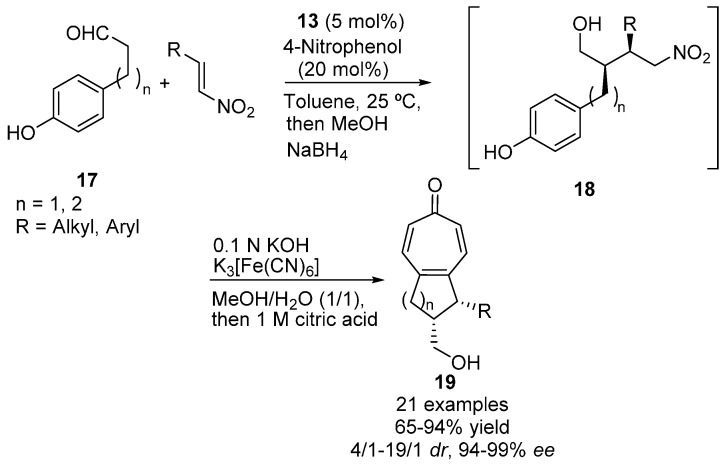
Asymmetric synthesis of *cis*-disubstituted dehydroazulenones using organocatalyst **13**.

**Scheme 6:**
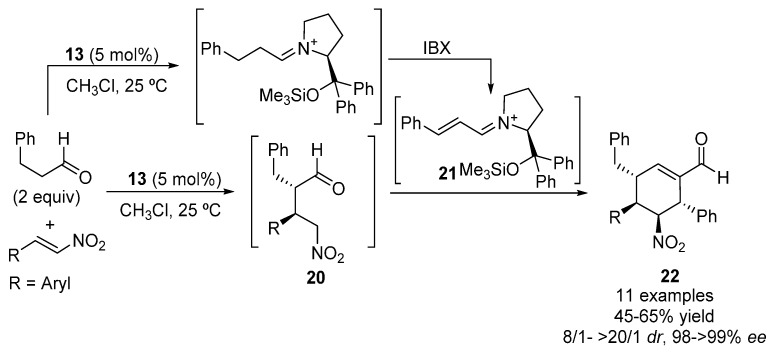
Asymmetric cascade reaction leading to cyclohexenes organocatalyzed by **13**.

In addition, this organocatalyst **13** has been applied to the preparation of the core structure of *Cephalotaxus* norditerpenes, with interesting antineoplastic and antiviral properties. Thus, phenol moiety-containing aldehydes **17** were asymmetrically added to β-arylated *trans*-nitroalkenes in the presence of the organocatalyst **13** and 4-nitrophenol as acid additive to give the corresponding nitroaldehydes, which were reduced in situ affording nitroalcohols **18**. Subsequent oxidative dearomatization gave *cis*-disubstituted dihydroazulenones **19** (Scheme 5) [41]. This organocatalyst **13** has also been used in an asymmetric cascade reaction leading to polyfunctionalized cyclohexene aldehyde derivatives in the presence of an oxidant such as *o*-iodoxybenzoic acid (IBX) [42]. Thus, Michael addition reaction of dihydrocinnamaldehyde (2 equiv.) with nitroalkenes led to intermediate nitroaldehydes **20** (Scheme 6). Simultaneously, the initial imine is oxidized in the presence of IBX to a conjugated iminium ion **21** which reacts as a Michael acceptor with the former adduct **20** to yield a cyclized product **22** through aldol condensation and hydrolysis. In addition, the same organocatalysts, in the presence of *p*-nitrophenol as co-catalyst, has been employed in the asymmetric reaction of succinaldehyde with 3-bromo-2-fluoronitrostyrene, to give a key intermediate in the total synthesis of the prostaglandin-analog beraprost [43]. Moreover, γ-nitroaldehydes type **2** have been prepared organocatalytically by means of **13**, and have been used for the synthesis of enantioenriched five-membered cyclic nitrones after reductive cyclization [44].

**Figure 3:**
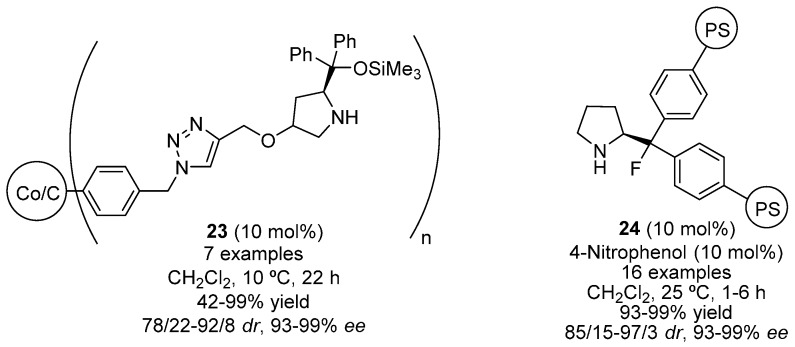
Supported organocatalysts for the asymmetric Michael addition of aldehydes to nitroalkenes leading to nitroaldehydes **2**.

**Figure 4:**
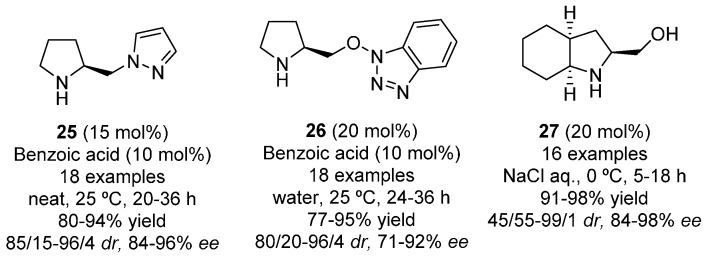
Results obtained using chiral pyrrolidine-containing organocatalysts in the asymmetric addition of aldehydes to nitroalkenes leading to nitroaldehydes **2**.

Some recovery-intended supported versions of this organocatalyst have been developed recently for the asymmetric addition of aldehydes to nitroalkenes. Thus, the organocatalyst has been grafted onto the surface of magnetic, polymer-coated cobalt/carbon nanobeads giving species **23**, which organocatalyzed the Michael addition of aldehydes to β-nitrostyrenes leading to γ-nitroaldehydes **2** (R^1^ = alkyl; R^2^ = alkyl, aryl) (Figure 3) [45]. The catalyst was magnetically separated, but its activity significantly decreased from the third run. However, the catalyst with a similar appendage has been grafted onto phosphorous dendrimers, the third generation displaying excellent recycling abilities since it could be recovered and reused in seven consecutive runs without loss of activity [45]. Moreover, a related fluorinated organocatalyst immobilized on a polystyrene resin **24** (Figure 3) has also been employed for the preparation of **2** (R^1^ = alkyl, R^2^ = aryl) [46]. The catalyst could be reused eight times, a decrease in its activity being observed after the seventh run. This supported catalyst has also been implemented in continuous flow processes. Furthermore, an ionically-tagged diphenylprolinol silyl ether has been employed in three different Michael additions of aldehydes to nitroolefins in ionic liquids, although its recyclability was very limited [47], similar to when a prolinol methyl ether carrying two perfluorohexylethyl groups was used as catalyst to give **2** (R^1^ = alkyl, R^2^ = alkyl, aryl), being recovered in only 40% by fluorous reverse-phase silica gel [48].

l-Proline has also been the chiral starting material for the preparation of other chiral organocatalysts employed in the asymmetric Michael additions of aldehydes to nitroolefins. Thus, the pyrrolidine-pyrazole compound **25**, combined with benzoic acid as additive, organocatalyzed the conjugate addition of aldehydes to β-aromatic *trans*-nitroalkenes, in absence of any solvent, giving rise to *syn*-γ-nitroaldehydes **2** (R^1^ = alkyl, R^2^ = aryl) (Figure 4) [49]. In addition, the pyrrolidine **26**, containing a hydroxybenzotriazole (HOBt) moiety, has shown to organocatalyze this reaction in water as solvent, the presence of benzoic acid being also necessary (Figure 4) [50]. Other proline-related catalyst is perhydroindolinol **27**, which produces the same asymmetric reaction driving to **2** (R^1^ = alkyl, R^2^ = aryl, alkyl), but using brine as solvent (Figure 4) [51].

**Scheme 7:**
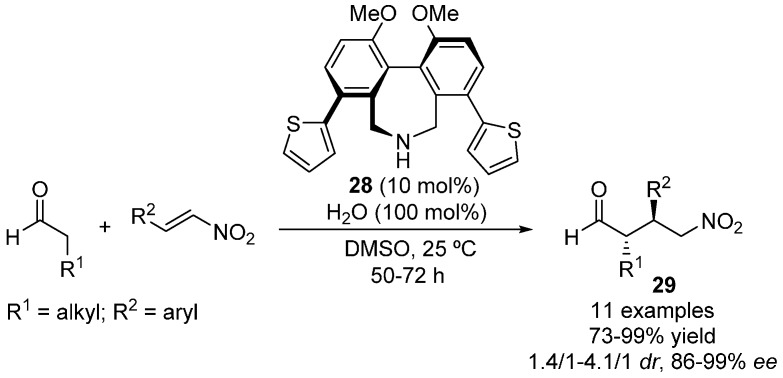
Asymmetric *anti*-selective Michael addition of aldehydes to nitroalkenes.

As previously shown, *syn*-nitroaldehydes of the type **2** are the main adducts obtained in the conjugate addition of aldehydes to *trans*-β-nitroalkenes. However, a less common *anti*-selectivity can be prevalent if some specific chiral amines are used as organocatalysts. Thus, the biphenyl-based chiral secondary amine **28** organocatalyze the Michael addition of aldehydes to nitrostyrenes, leading mainly to the less accessible *anti*-adducts **29** (Scheme 7) [52].

**Scheme 8:**
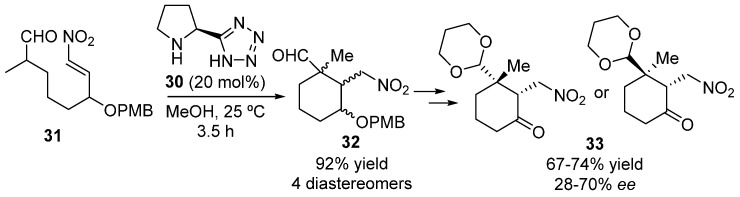
Asymmetric synthesis leading to the atropurpuran A-ring.

The asymmetric synthesis of the A-ring fragment of C19-diterpenoid atropurpuran has been achieved after a key step consisting of an intramolecular Michael addition reaction using 5-pyrrolidinyl tetrazole **30** as organocatalyst (Scheme 8). In this way, nitroalkene **31** afforded four diastereomeric cyclohexanes **32** which were converted into two substituted cyclohexanones **33**, with different enantioselectivities according to the starting diastereomer [53].

**Scheme 9:**
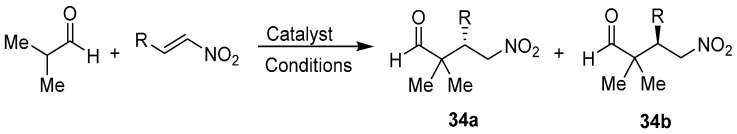
Asymmetric organocatalytic Michael addition of isobutyraldehyde to nitroalkenes.

**Figure 5:**
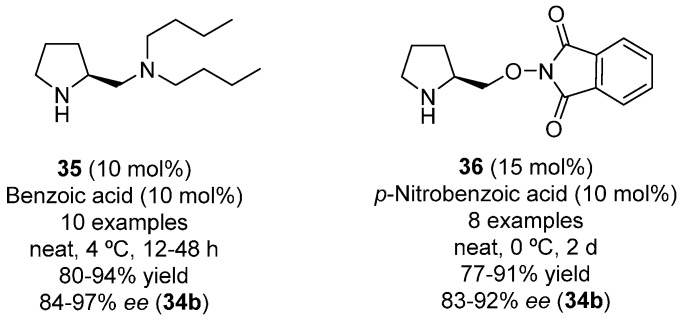
Results obtained using chiral pyrrolidine-containing organocatalysts in the asymmetric addition of isobutyraldehyde to nitroalkenes leading to nitroaldehyde **34b**.

The Michael addition of α,α-disubstituted aldehydes to nitroalkenes provides adducts possessing all-carbon quaternary centers. Isobutyraldehyde is an adequate aldehyde for this organocatalytic asymmetric reaction (Scheme 9), which justifies its frequent use as a model when a new organocatalyst is developed. Some examples of chiral pyrrolidine-containing catalysts are diamine **35** [54] and pyrrolidine-oxyimide **36** [55], which led to enantiomer **34b** (R = aryl) under solvent-free conditions and in the presence of a carboxylic acid as co-catalyst (Figure 5). The same enantiomer was mainly obtained using the pyrrolidine-HOBt **26** as organocatalyst (20 mol %), in water as solvent at 0 °C and in the presence of benzoic acid as additive (10 mol %) (73–91% yield, 77–93% *ee*) [50]. This three organocatalysts have also been employed using cyclopentanecarbaldehyde as a pro-nucleophile affording the compound similar to **34b** [(CH_2_)_4_ instead of 2Me] (using **35**: 76–93% yield, 92–96% *ee*; using **36**: 80–91% yield, 78–92% *ee*; using **26**: 69–91% yield, 74–92% *ee*).

**Figure 6:**
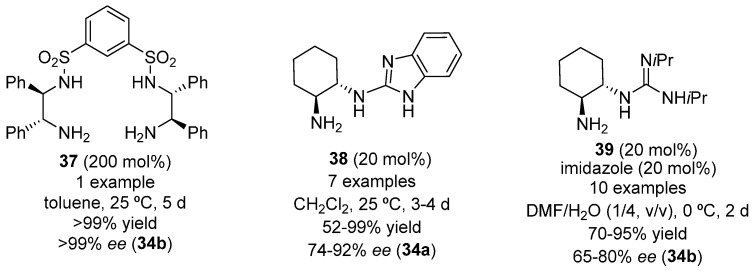
Results obtained using C2-diamine-derived organocatalysts in the asymmetric addition of isobutyraldehyde to nitroalkenes leading to nitroaldehydes **34**.

C2-Symmetric diamines have been employed profusely as chiral blocks in the preparation of organocatalysts for asymmetric reactions [56]. An example is the use of wedge shaped 1,3-benzenedisulfonamide **37** (Figure 6), obtained from (1*R*,2*R*)-1,2-diphenylethane-1,2-diamine, in the Michael addition of isobutyraldehyde to β-nitrostyrene leading to **34b** (R = Ph) [57]. This compound **37** mimics the chiral pocket of an enzyme, embedding the nitro group in the groove of the catalyst by means of double hydrogen bonding, whereas the enamine from the aldehyde is formed with one of the primary amino groups. However, chiral *trans*-cyclohexa-1,2-diamines have been the C2-symmetric diamines which have been more frequently used when designing new chiral organocatalysts. An example is the primary amine-containing benzimidazole **38** (Figure 6), which acts bifunctionally by hydrogen-bond coordination with one of the hydrogens of the nitro group of the nitroalkene, while the last reacts with the primary amine-formed enamine [58]. Another example is the guanidine-containing primary amine **39** (Figure 6), which has been used for the asymmetric conjugate addition of isobutyraldehyde to nitrostyrenes to give preferentially **34b** (R = aryl), working in an aqueous medium [59]. In this case the catalyst cannot be considered as bifunctional, as it has been determined by theoretical calculations that, probably due to the presence of water, the guanidine function is not coordinated to the nitro group in the transition state.

**Figure 7:**
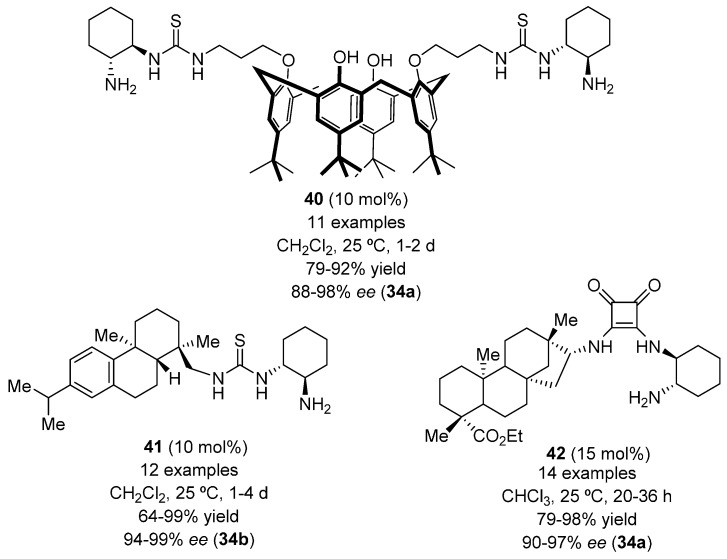
Results obtained using primary-amine thioureas and squaramides as organocatalysts in the asymmetric addition of isobutyraldehyde to nitroalkenes leading to nitroaldehydes **34**.

Chiral organocatalysts bearing a primary or a secondary amine and a thiourea have proven quite effective in many asymmetric transformations involving aldehydes or ketones [60,61,62,63]. Concerning acidity and geometric distances, the hydrogens on the thiourea group can hydrogen bond to both oxygens of the nitro group in the nitroolefin. Examples of organocatalysts recently employed in the asymmetric Michael addition of isobutyraldehyde to nitrostyrenes are shown in Figure 7.

**Scheme 10:**
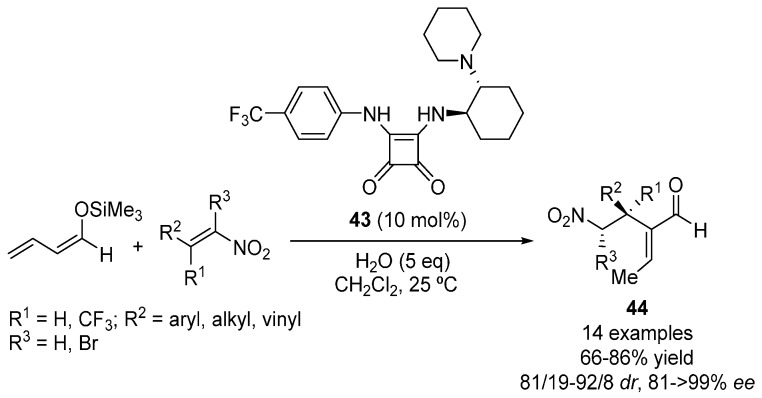
Asymmetric organocatalytic addition of aldehyde-derived silyl-enol ethers to nitroalkenes.

Thus, the use of calyx[4]arene-based chiral bifunctional primary-amine thiourea **40** has led to the adduct **34a** (R = aryl) [64], whereas the use of rosin-based primary-amine thiourea **41** drove to **34b** (R = aryl) in high yields and enantioselectivities [65]. This last organocatalyst **41** has also been used in the addition of other differently α,α-disubstituted aldehydes to β-nitro-4-nitrostyrene, achieving highs *ee*’s (up to 99%) of the final nitroaldehyde, although with moderate diastereo selectivities (up to 83/17). Kinetic and spectroscopic studies of the conjugate addition of 2-phenylpropanal to nitroolefins catalyzed by two different primary amine thiourea catalysts have shown that, at least in these cases, the resting state is a product imine species that undergoes reversible hydrolysis to form the reaction product, and that reversibility of the reaction is implicated in cases of low enantio- and diastereoselectivity [66]. Amine-containing squaramides have shown as efficient bifunctional organocatalysts in many asymmetric processes as amine-containing thioureas [67,68]. A recent example is the use of isosteviol-containing primary amine-squaramide **42** as organocatalyst in the asymmetric conjugate addition of isobutyraldehyde to nitroalkenes, achieving adduct **34a** (R = aryl, alkenyl) (Figure 7) [69]. It is interesting to note that similar values of the opposite final configuration **34b** has been achieved exchanging the cyclohexa-1,2-diamine moiety of the catalyst by its corresponding enantiomer, showing that the isosteviol moiety is not involved in the enantioselectivity. Moreover, the regioselective functionalization of aldehyde-derived silyl−diene enol ethers under a bifunctional tertiary amine-containing squaramide organocatalyst **43** has shown to produce a change in the regioselectivity, from the 1,5- to the 1,3-functionalization, affording Rauhut-Currier-type conjugated aldehydes **44** after double bond isomerization, as exemplified in Scheme 10. This procedure has made possible the 1,3-addition of silyl−dienol ethers to nitroalkenes [70].

### 2.2. Ketones

**Scheme 11:**
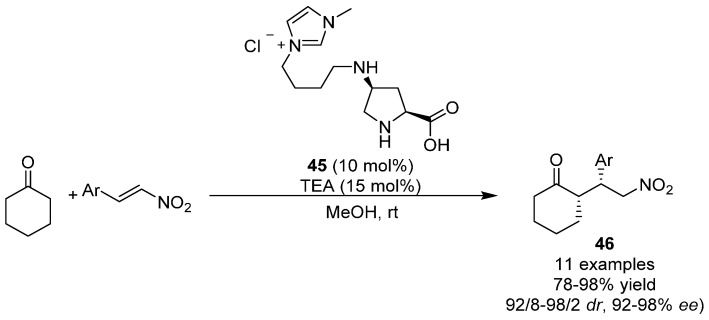
Asymmetric organocatalytic Michael addition of cyclohexanone to nitroalkenes organocatalyzed by ionic-liquid **45**.

Among ketones, cyclohexanone have been profusely used as a model pro-nucleophile to test the catalytic efficiency of organocatalysts in the Michael addition to nitroalkenes. Although high levels of diastereo- and enantioselectivities have been obtained with several organocatalysts in the addition of six-membered ring ketones to β-nitroolefins, other cyclic or acyclic ketones gave poorer results [8]. In this context, the conjugate addition of ketones to β-nitrostyrenes catalyzed by l-proline showed a strong solvent effect, giving the best results in MeOH [28,29,71]. This was the solvent of choice to perform the addition of cyclohexanone to several aryl nitroolefins catalyzed by chiral ionic liquid containing proline moiety **45** in the presence of triethylamine (TEA) as co-catalyst (Scheme 11), leading to adducts **46** [72]. The re-usability of the catalyst was proven, as similar results were achieved over five reaction runs after catalyst recovering from the reaction media by precipitation with diethyl ether.

Some proline derivatives that have shown their efficiency as organocatalysts in the reaction between aldehydes and β-aromatic *trans*-nitroalkenes, were also tested under similar reactions conditions in the related process with ketones. Thus, pyrrolidine **26** (10 mol %) containing a HOBt moiety (Figure 4) promoted the Michael addition of cyclohexanone to aryl nitroolefins in water as solvent, providing the corresponding γ-nitrocarbonyl derivatives **46** in good yields (84–95%), with high diastereo- (91/9–96/4) and enantioselectivities (81–95%) irrespective of the substitution pattern on the nitroolefins. However, lower results were achieved with other ketones, such as tetrahydro-*4H*-pyran-2-one, cyclopentenone or acetone [73]. Similar results were encountered when organocatalyst **36** (10 mol %) containing a oxyimide appendage (Figure 5) was used in water for the Michael addition of cyclic ketones as pro-nucleophiles and aromatic *trans*-nitrostyrenes (86–95% yield, 91/9–97/3 *dr*, 85–96% *ee*). However, the reaction with acetone was very sluggish and proceeded with low stereoselectivity [74]. For both catalysts, the use of additives was ruled out since the effective hydrogen bonding interaction provided by the water molecules brings closer both substrates with the appropriate orientation, which would be less effective in the presence of an additive.

**Figure 8:**
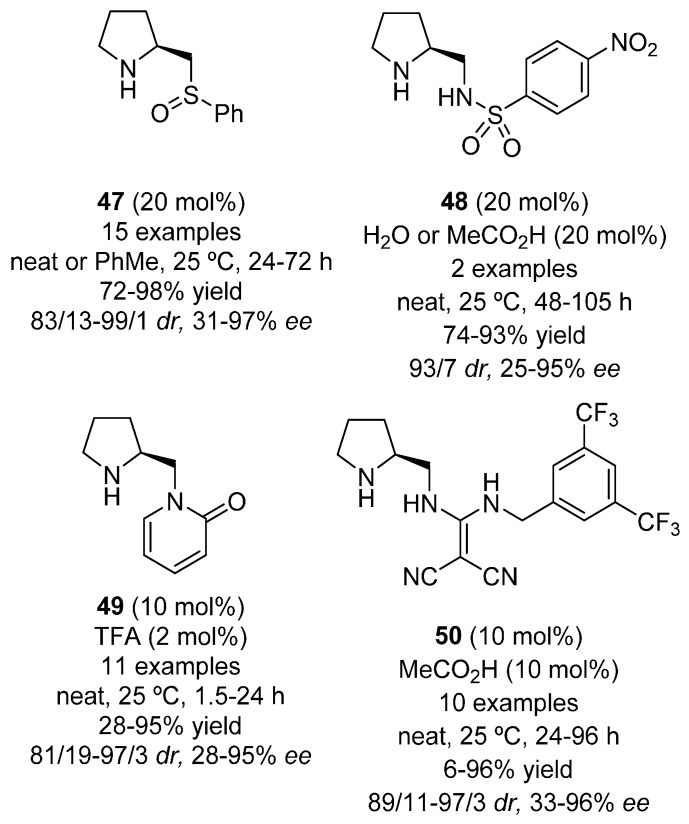
Results obtained using some pyrrolidine-based organocatalysts in the Michael reaction of ketones to β-nitrostyrenes.

Several pyrrolidine-based catalysts containing a functional group in the side chain that provided an additional hydrogen-bond interaction have been developed and tested in this transformation. Thus, pyrrolidine derivative **47** (Figure 8) bearing a sulfoxide moiety was very effective in the addition of cyclohexanone, cyclopentanone and acetone to aromatic β-nitroolefins [75]. However, lower enantioselectivities (31–51% *ee*) were achieved when 2-butanone and 3-pentanone were used. Remarkably, the formation of the Michael adduct took place at the more substituted site when using 2-butanone.

The addition of a sulfonamide moiety to the pyrrolidine side chain led to the catalyst **48** which organocatalyzed the addition of cyclohexanone and acetone to *trans*-β-nitrostyrene under neat conditions. The presence of water or acetic acid as additives improved the achieved results with cyclohexanone (concerning yield and enantioselectivity), whereas the opposite effect was observed with acetone [76].

Pyrrolidine-pyridone catalyst **49** was designed to achieve high results in diastereo- and enantioselectivities due to the effective shield of one side of the enamine intermediate, with the pyridine moiety. Also, the carbonyl group of the ring could stabilize the transition state through hydrogen bonding interactions. This catalyst was tested in the addition of cyclohexanone to aryl and heteroaryl nitroolefins under neat conditions affording products **46** with good results, in terms of yields and stereoselectivities. Similar result was obtained when tetrahydro-4*H*-pyran-2-one was used as reagent, while acyclic ketones, such as acetone or 2-butanone, gave poorer results [77].

A double hydrogen bonding group was introduced in the pyrrolidine catalyst by using a diamino methylene malononitrile skeleton at the side chain (Figure 8, **50**). Thus, catalyst **50** in the presence of acetic acid as additive under neat conditions, gave the corresponding Michael adducts in the reaction of cyclohexanone with aromatic and heteroaromatic nitroalkenes with good results. However, the use of other ketones such as acetone led to disappointing results in terms of enantioselectivity (33%) [78].

**Figure 9:**
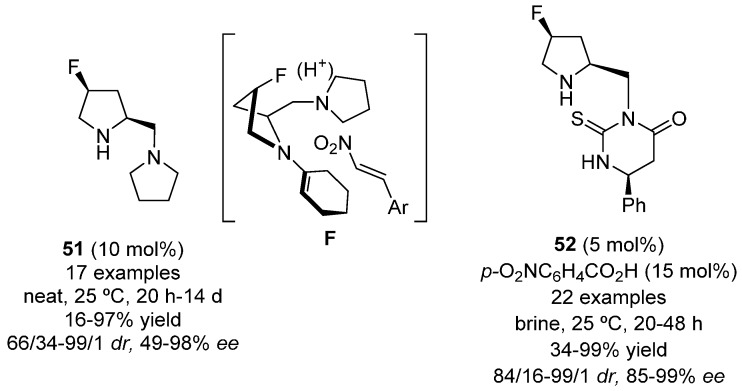
Pyrrolidine-based organocatalysts containing a fluorine atom at the ring in the Michael addition of ketones to nitroalkenes.

In order to study the effect of the conformation of the pyrrolidine ring on the outcome of the reaction, a fluorine atom was added at the 4-position of the pyrrolidine ring in catalyst **51** (Figure 9). The gauche effect cause by the fluorine atom led to a drastic improvement in the formation of products of type **46**, under solvent free conditions. It was observed that the addition of 10 mol% of trifluoroacetic acid provided better stereoselectivities when electron deficient substituted β-nitrostyrenes were used as electrophiles, while the contrary occurred for their electron-rich substituted counterparts. Furthermore, the catalyst was quite efficient in promoting the reaction of other cyclic ketones and acetone with nitrostyrenes [79]. As shown in the proposed stereochemical model **F** (Figure 9) created by reaction with cyclohexanone, when a fluorine atom was introduced at the 4-position of the pyrrolidine ring, the (*E*)-*trans*-C^γ^-*endo* is the favored conformation due to the gauche effect.

This gauche effect was also observed in catalyst **52**, the *cis*-diastereoisomer providing the best results. The introduction of the fluorine atom at the 4-position of the pyrimidinone substituted pyrrolidine ring improved not only the yield but also the stereoselectivity, an allowed to reduce the catalyst loading to 5 mol %. Not only aromatic nitroalkenes with electron-withdrawing and electron-donating substituents were suitable electrophiles, but also some aliphatic nitroalkenes and aromatic nitrodienes were successfully used as Michael acceptors. The use of brine and the possibility of reducing the reagent ratio to almost stoichiometric values by using catalyst **52** made this protocol green and effective [80].

**Figure 10:**
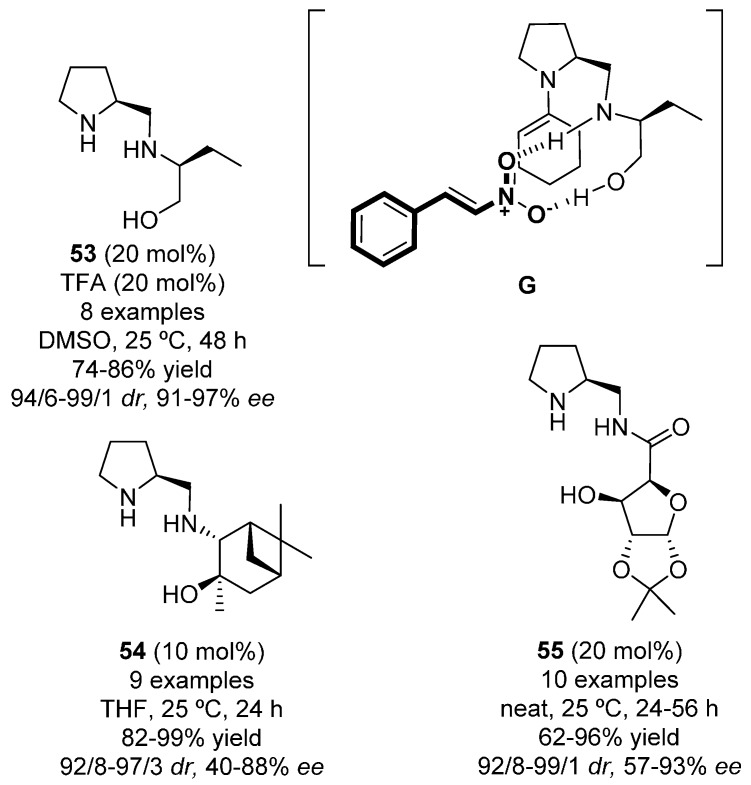
Pyrrolidine-based organocatalysts containing an additional stereogenic center in the conjugate addition of ketones to nitroolefins.

Another way of trying to improve the results of the pyrrolidine-based catalyst in the Michael reaction of ketones to nitroolefins was to introduce an additional stereogenic center in the side chain, as well as a new hydrogen-bonding functionality. Thus, chiral 1,2-amino alcohols have been incorporated to the pyrrolidine moiety producing bifunctional organocatalysts such as compound **53** (Figure 10). This catalyst, in the presence of trifluoroacetic acid, gave good results in the reaction between cyclohexanone and β-nitrostyrene derivatives. DFT calculations showed that both NH and OH groups activated the nitro moiety by the formation of hydrogen bonds, as illustrated in the coordination model **G**, favoring its approach from the *Re*-face of the *anti*-enamine [81].

Natural pinane-derived bifunctional catalyst **54** promoted also effectively the reaction between cyclohexanone and heterocyclohexanone derivatives to aromatic and heteroaromatic nitroolefins. But the reaction gave either poor enantioselectivity (40%) with acetone and failed with cyclopentanone or 1,4-cyclohexanedione monoethylene-ketal [82]. In addition, better results and substrate scope were achieved by using sugar amide-derived catalyst **55** (Figure 10) under solvent-free conditions. Several β-nitrostyrenes were used as electrophiles in the reaction with cyclohexanone, providing good results irrespective of the nature of substitution at the aromatic ring. Also cyclopentanone and acetone were suitable pro-nucleophiles, although the achieved results, in terms of yields and selectivities, were lower compared to those obtained with standard cyclohexanone [83].

**Figure 11:**
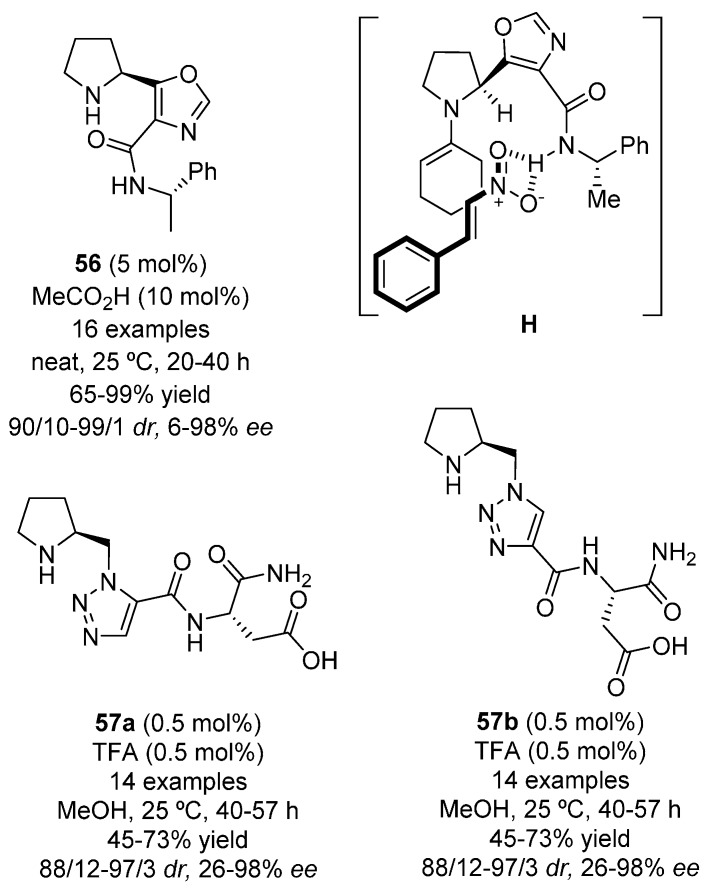
Pyrrolidine-based organocatalysts containing an additional stereogenic center linked by a heterocyclic ring.

The introduction of a heterocyclic linkage between the pyrrolidine ring and the unit bearing an additional stereogenic center has been tested to study if the new rigid backbone conformation provided by the heterocycle would enhance the stereoselectivity (Figure 11). Thus, catalyst **56** [84], **57a**,**b** [85] have been used in the reaction between ketones and aryl nitroolefins under different reaction conditions, providing adducts of type **46** in good results. Lower yields and enantioselectivities were found when acetone was used as pro-nucleophile. Remarkably, only 0.5 mol % of catalyst loading for compounds **57** was enough to promote the reaction, independently of the relative position of amide in the heterocycle. DFT calculations for catalyst **56** showed that the intramolecular hydrogen bonds and the steric hindrance of the *N*-benzyl oxazolecarboxamide moiety control the *Si*-face approach and the catalytic activity, as illustrated in the coordination model **H** (Figure 11).

**Figure 12:**
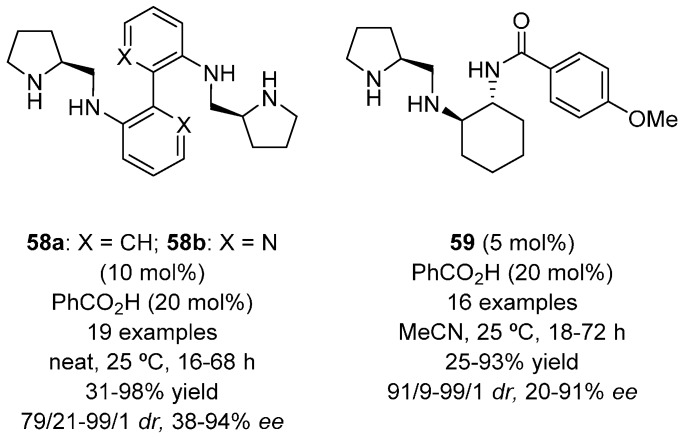
Results obtained using pyrrolidine-based organocatalysts with a diamine backbone in the preparation of nitroketone **46**.

Diamines have also been employed as backbone to anchor pyrrolidine units to synthetize useful organocatalysts (Figure 12). Examples of this type of derivatives are catalysts **58** (**a**: X = CH; **b**: X = N) [86] and **59** [87]. Both systems have been used in combination with benzoic acid in the standard Michael addition of cyclohexanone to several aryl and heteroaryl nitrostyrenes.

**Scheme 12:**
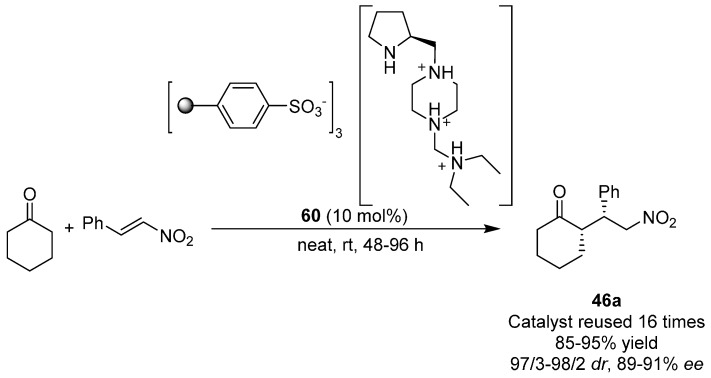
Michael addition of cyclohexanone to β-nitrostyrene organocatalyzed by resin- immobilized **58**.

Some efforts destined to recycle pyrrolidine-based catalyst active in the Michael addition of cyclohexanone to *trans*-β-nitrostyrene have been carried out. Thus, resin-immobilized pyrrolidine catalyst **60** [88] has been used, under solvent free conditions, in this transformation for 16 successive runs without significant loss of enantioselectivity (Scheme 12). The catalytic efficiency was also demonstrated by using other nitroolefins as electrophiles, leading to similar results in terms of yields and stereoselectivities. However, cyclopentanone, cycloheptanone or propanal gave the corresponding Michael adducts with moderate enantioselectivities (15–50%).

**Figure 13:**
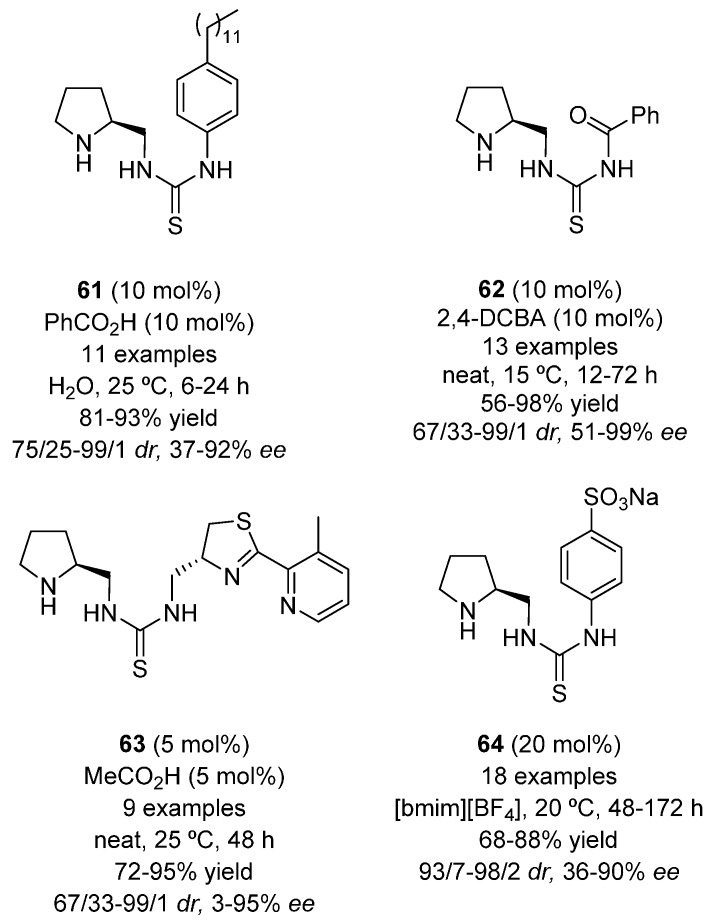
Results obtained using pyrrolidine-based thiourea organocatalysts in the Michael addition of ketones to nitroalkenes.

Privileged type of organocatalysts used in this Michael addition are those containing a thiourea moiety. Examples of the combination of the pyrrolidine ring together with the thiourea group are shown in Figure 13. Thus, the surfactant-type thiourea **61** worked smoothly in water as solvent, giving adducts of type **46** in good results. When the same reaction conditions were applied to cyclopentanone or acetone, the achieved enantioselections were lower [89]. A similar trend was observed when catalyst **62** was used in the presence of 2,4-dichlorobenzoic acid (2,4-DCBA) for the Michael addition of ketones to β-nitroolefins under neat conditions. Although high enantioselectivities were achieved in the reaction of cyclohexanone with aromatic and, even, aliphatic nitroolefins, results were not satisfactory when those conditions were applied to cyclopentanone or acetone [90]. The same catalyst **62** was also able to promote the addition of cyclohexanone to β-nitrostyrenes in water, but in the presence of 2-nitrobenzoic acid (10 mol %), which enhanced the obtained yields [91]. Poor results were also obtained when acetone was used as pro-nucleophile with *trans*-nitrostyrene using thiazoline-thiourea-pyrrolidine catalyst **63** under neat conditions. However, these reaction conditions allowed the preparation of adducts **46** in good results [92]. The ionic liquid-soluble pyrrolidine-urea **64** promoted the Michael addition of cyclohexanone or acetone to several aryl and heteroaryl β-nitroolefins. Although the yields were constantly high for both ketones, the enantioselectivities were lower for adducts arising from acetone. This catalytic system could be reused four times with comparable results [93].

**Scheme 13:**
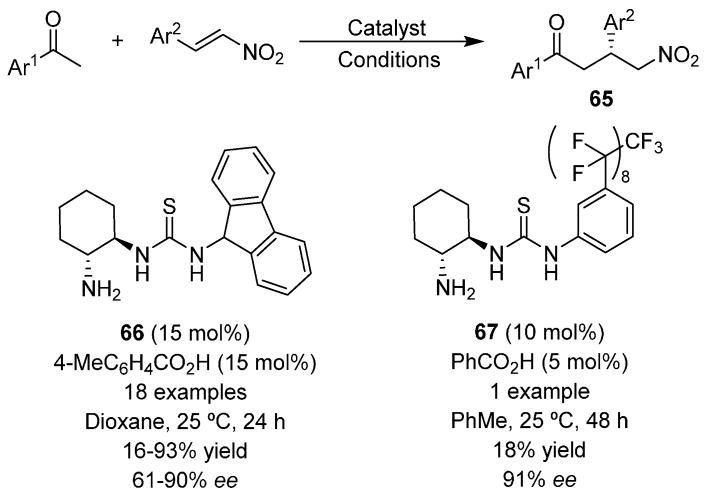
Michael addition of aryl methyl ketones to nitrostyrenes organocatalyzed by chiral cyclohexane-1,2-diamine-thiourea derivatives.

The combination of the thiourea motif with *trans*-1,2-diaminocyclohexane as the chiral unit, has led to organocatalysts able to catalyze the Michael addition of acetophenone derivatives to β-nitrostyrenes (Scheme 13). Similarly to the case of aldehydes, in this type of catalysts the primary amine activates the carbonyl compound via iminium or/and enamine formation, whereas the thiourea activates the electrophile via hydrogen bonding. In catalyst **66**, the sterical hindrance provided by fluorenyl backbone allowed the formation of adducts **65** with moderate to good enantioselectivities [94]. The introduction of a perfluorated alkyl chain in this type of catalyst led to compound **67**, which was tested in the Michael addition of acetophenone to *trans-*β-nitrostyrene with low yields albeit in excellent enantioselectivity [95].

**Scheme 14:**
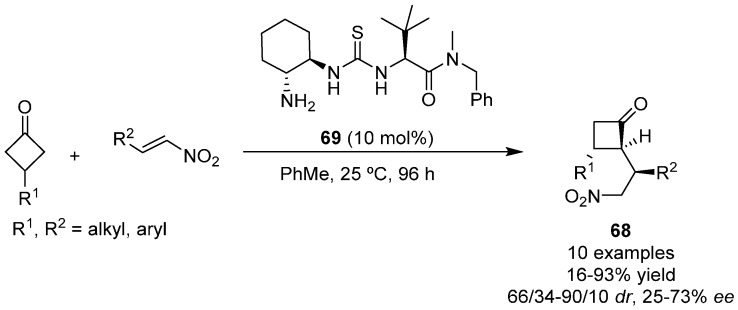
Desymmetrization of prochiral 3-substituted cyclobutanones organocatalyzed by **67**.

The thiourea-amino acid catalyst **69** has been used to perform the desymmetrization of prochiral 3-substituted cyclobutanones after Michael addition to *trans-*β-nitrostyrene derivatives, leading to products **68** with moderate enantioselectivities, and with one predominant isomer of the possible diastereoisomers (Scheme 14) [96].

**Scheme 15:**
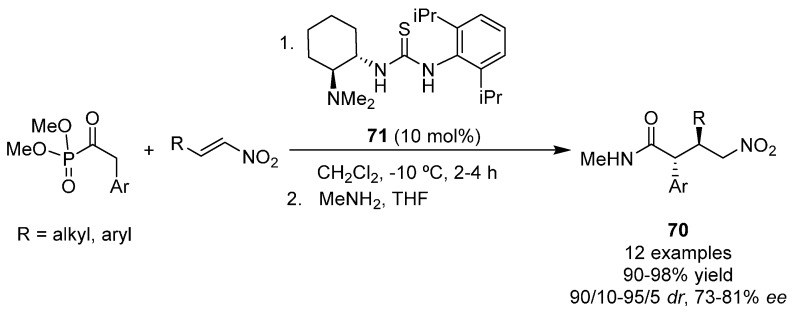
Michael addition of β-aryl-α-ketophosphonates to nitroalkenes organocatalyzed by thiourea **69**.

A thiourea derived from cyclohexane-1,2-diamine **71** has shown to be an efficient catalyst to carry out the Michael reaction of unusual β-aryl-α-ketophosphonates to nitroalkenes giving products **70** with excellent diastereoselectivities and good enantioselectivities (Scheme 15). In this case, the tertiary amine moiety of catalyst **71** deprotonates the β-aryl-α-ketophosphonate leading to an enolate which is closely associated with the protonated catalyst by ionic and hydrogen bonding interactions [97].

**Scheme 16:**
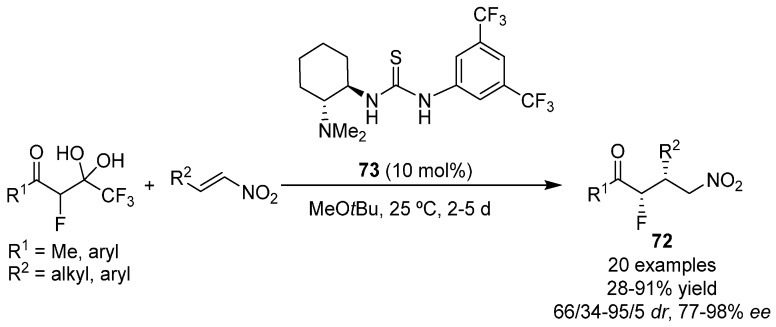
Michael addition of trifluoromethyl α-fluorinated *gem*-diols to nitroolefins organocatalyzed by Takemoto’s thiourea organocatalyst.

The Takemoto’s thiourea catalyst **73** has allowed the synthesis of γ-nitro-α-fluorocarbonyl compounds **72** with good results (Scheme 16). In this transformation, after the initial formation of the primary asymmetric Michael adduct, the amino group of the catalyst abstracts a proton from the addition intermediate, releasing a trifluoroacetate unit. Then, the enolate is asymmetrically re-protonated by the ammonium catalyst to give compounds **72** [98].

The same catalyst **73** was used in the addition of 1,3-bis(phenylthiol)propa-2-one to nitrostyrenes, affording the expected products (six examples) with moderate yields (74–91%), diastereoisomeric ratios from 43/57 to 67/33 and enantioselectivities in the range 69–81% [99].

**Scheme 17:**
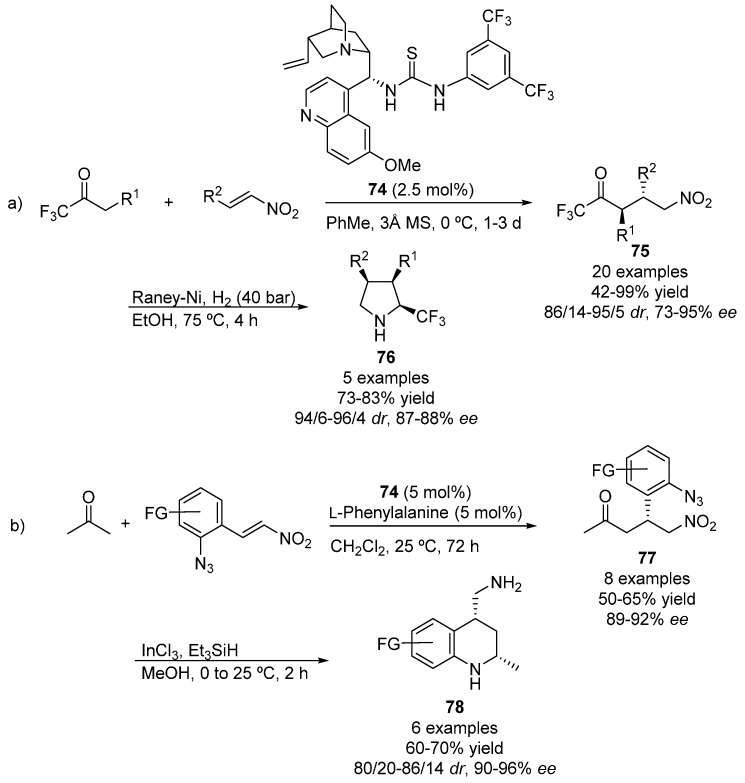
Sequential Michael addition-diastereoselective amino reductive cyclization processes.

The combination of amines derived from *Cinchona* alkaloids with the thiourea moiety has led to several organocatalysts useful for different transformations. For example, catalyst **74** has been used in the Michael addition of trifluoromethyl ketones to nitroolefins (Scheme 17a). After diastereoselective amino reductive cyclization of compounds **75**, the corresponding pyrrolidines **76** were isolated with good yields and stereoselectivities [100]. In addition, the use of the same catalyst **74** in combination of l-phenylalanine organocatalyzed the reaction of acetone with different substituted azido nitrostyrenes to give the expected compounds **77** with moderated yields and good enantioselectivities (Scheme 17b). The diastereoselective amino reductive cyclization using indium salts and silanes allowed the preparation of tetrahydroquinolines **78** with moderate yields and high stereoselectivities [101].

The versatility of this catalyst **74** has been also showed in the simple Michael addition of cyclopentane-1,2-dione to different nitrostyrenes (11 examples), affording the expected adducts with yields ranging from 60 to 90% and moderate enantioselectivities (56–76%) [102].

**Scheme 18:**
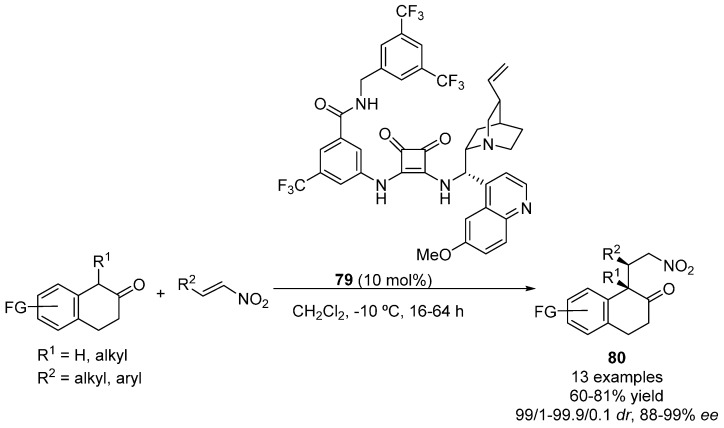
Asymmetric organocatalyzed Michael addition of β-tetralones to nitroolefins.

A related squaramide-*Cinchona* Brønsted-base organocatalyst **79** has been developed and tested in the Michael addition of α-monosubstituted β-tetralones to nitroalkenes (Scheme 18). The reaction also took place using non-substituted tetralones and other aromatic ring-fused cycloalkanones as pro-nucleophiles, providing the corresponding products in good results (80–88% yield, 50/50-95/5 *dr*, 99% *ee*). The obtained adducts of type **80** were converted into diverse polycyclic compounds bearing up to six stereogenic centers, including spirocyclic structures [103].

**Scheme 19:**
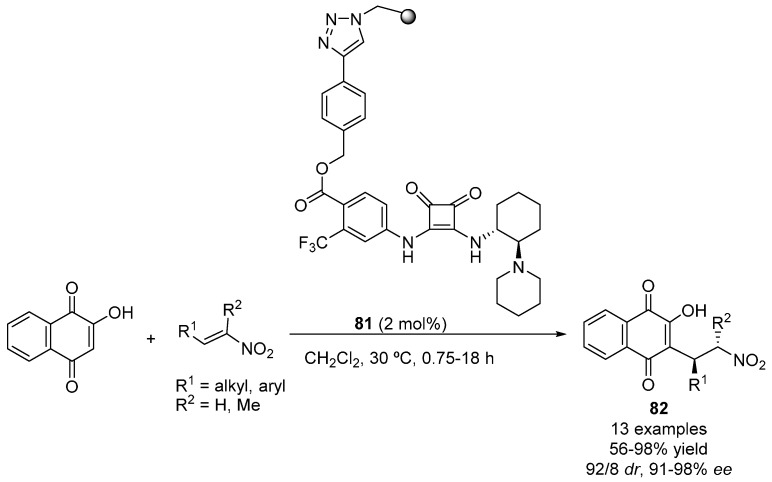
Organocatalyzed Michael addition of 2-hydroxy-1,4-naphthoquinone to nitroolefins under continuous flow chemistry.

The possibility of using continuous flow chemistry using organocatalysts has also been exemplified by using a polystyrene-supported squaramide derived from chiral *trans*-cyclohexa-1,2-diamine **81** as organocatalyst in the Michael addition of 2-hydroxy-1,4-naphthoquinone to nitroalkenes (Scheme 19).

Thus, the β-dicarbonyl naphthoquinone derivative reacted with β-nitrostyrenes bearing electron-withdrawing or -donating groups, as well as 2-nitro-1-heteroarylethylene derivatives, lead to the corresponding products **82** in good yields and enantioselectivities. Catalyst **81** was recovered and reused for 10 consecutive runs without detrimental effects, and adapted to a continuous flow operation (24 h), achieving a high accumulated TON of 200 and a productivity of 4.07 mmol/g resin h [104].

A single flow experiment involving six different nitroalkenes in a sequential manner showed the applicability of the system for the preparation of small libraries of enantiopure compounds. A related polystyrene-supported squaramide organocatalyst has been used in a continuous flow process involving a sequential two-step transformation consisting of a squaramide-catalyzed Michael addition reaction of 2-hydroxy-1,4-naphthoquinone and acetoxylated nitroalkanes, and a subsequent oxa-Michael cyclization to give pyranonaphthoquinones [105].

**Figure 14:**
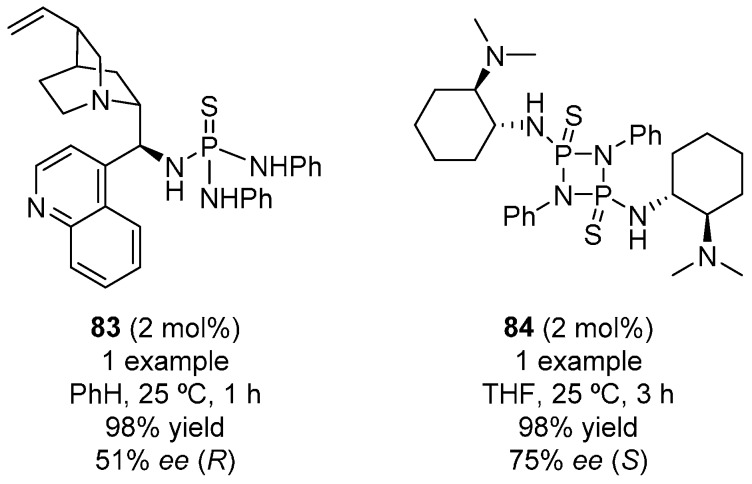
Phosphoroamide-type organocatalysts used in the Michael addition of 2-hydroxy-1,4-naphthoquinone to *trans-*β-nitrostyrene leading to **80**.

This transformation has also been performed using phosphoramide derivatives as organocatalyst (Figure 14). Thus, while the open-chain triamide **83** gave product **82** (R^1^ = H, R^2^ = Ph) in almost quantitative yield and moderate enantioselectivity, the *cis*-cyclodiphosphane derivative **84** led to the same product **82** with improved enantioselectivity but as the opposite enantiomer. These results have been confirmed by means of theoretical calculations, showing that open-chain phosphorous triamide complexes present a weaker ability to perform hydrogen bonds compared to cyclophosphane derivatives [106].

**Figure 15:**
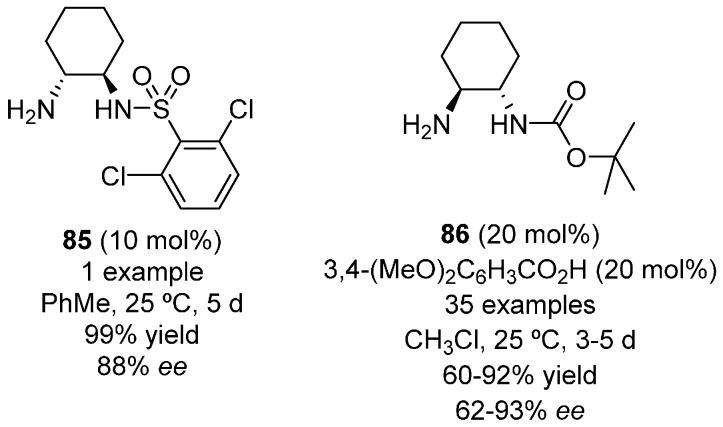
1,2-Diaminocyclohexane-based organocatalysts used in the Michael addition of acetone (**85**, **86**) and aryl methyl ketones (**86**) to *trans-*β-nitroolefins.

A weak halogen bond donor is involved in the transition state proposed for catalyst **85**. For this system, DFT calculations predicted a Cl-O interaction involving an oxygen atom of the nitro group and the chlorine of catalyst in the reaction between acetone and *trans-*β-nitrostyrene (Figure 15) [107].

A hydrogen bond interaction explained the stereochemical outcome achieved when catalyst **86** was used in the reaction of aryl methyl ketones with aryl and heteroaryl nitroolefins. In this case, computational studies showed that, in apolar solvents, the existence of the hydrogen bond explained the formation of the (*R*)-Michael product in high enantioselectivities whereas in polar solvents the partial rupture of the H-bond activation induces the formation of the (*S*)-product, explaining the loss of enantioselectivity [108].

**Scheme 20:**
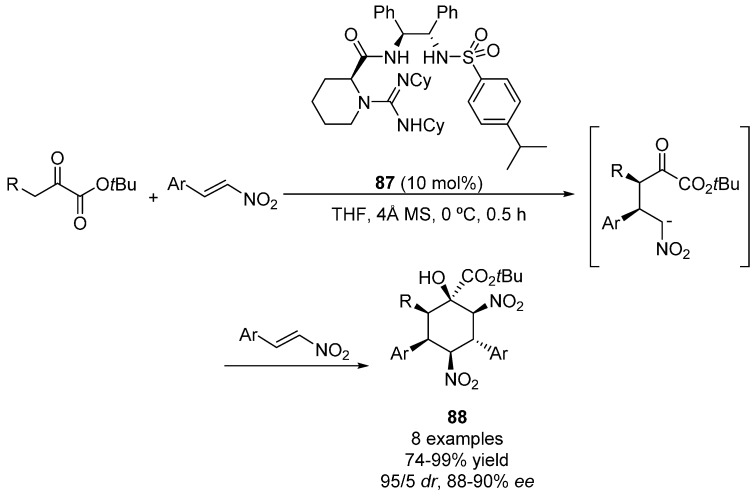
Construction of cyclohexanes with six vicinal stereogenic centers through a Michael/Michael/Henry process.

The cooperation between Brønsted base catalysis with the hydrogen bonding ability provided by the guanidine-sulfonamide catalyst **87**, promoted the deprotonation of α-keto esters, which reacted with two β-nitrostyrene derivatives in a triple cascade Michael/Michael/Henry process, allowing the formation of hexa-substituted cyclohexane derivatives **88**, with a quaternary stereocenter, in good yields and excellent stereocontrol (Scheme 20) [109].

**Figure 16:**
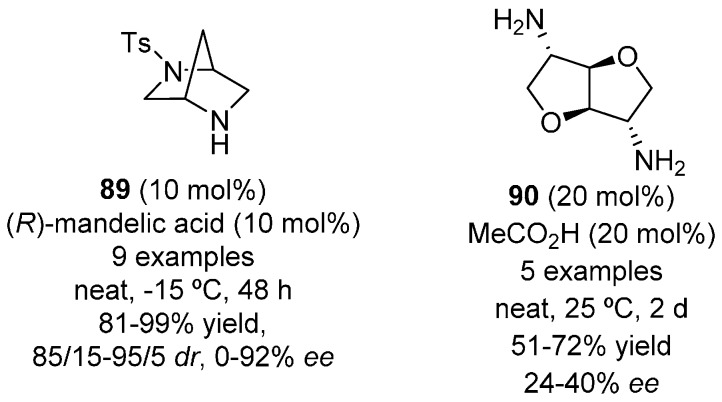
Simple chiral amine organocatalysts used in the Michael addition of ketones to *trans-*β-nitrostyrenes.

The combination of simple chiral amines with different structures bearing a Brønsted acid has served as promoters for the Michael addition of ketones to nitroalkenes (Figure 16). One example of this type of cooperative catalyst was observed in the reaction between cyclohexanone and nitroolefins catalyzed by the combination of chiral secondary amine **89** and (*R*)-mandelic acid, providing products **46** under neat conditions [110]. *ortho*-Substituted aryl nitroolefins led to good enantioselectivities, whereas lower results were observed when the substitution was located at the *para*-position. In addition, d-isomannide **90** combined with achiral acetic acid has been used to catalyze the addition of acetone and acetophenone to aryl β-nitroolefins, albeit in moderate results [111].

**Scheme 21:**
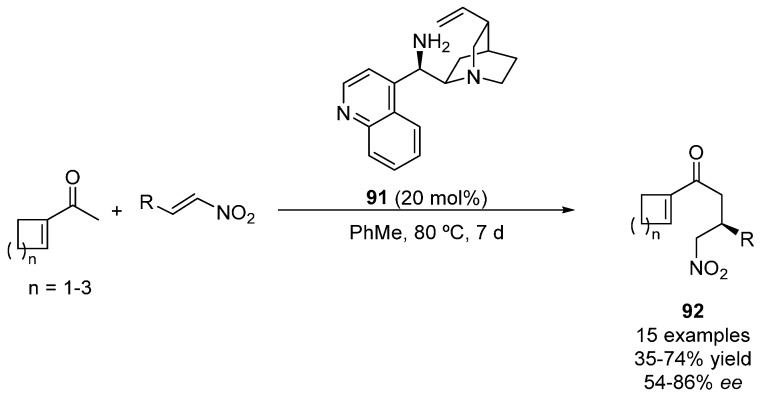
Asymmetric organocatalyzed addition of 1-acetylcycloalkenes to β-nitroolefins.

Finally, accessible amino-*Cinchona* derivatives combined to acid additives have been used as catalytic system for diverse Michael processes. Thus, *epi*-cinchonidine amine **91** was used as chiral organocatalyst in the addition of different 1-acetylcycloalkene derivatives to nitroalkenes, providing products **92** in moderate enantioselectivities (Scheme 21). Subsequent treatment of these products with tetramethylguanidine gave the expected bicyclic compounds, after a new Michael addition process [112].

**Scheme 22:**
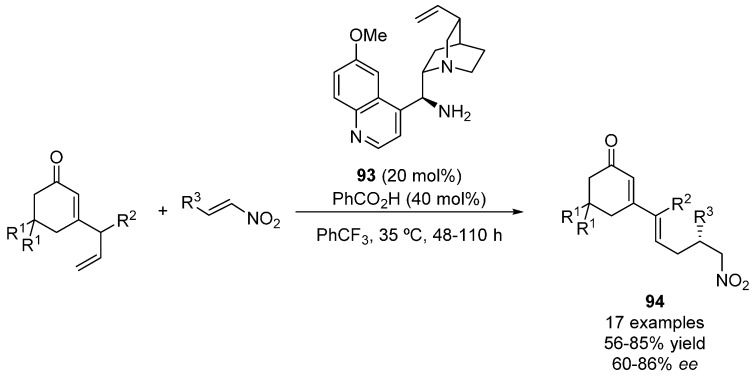
Organocatalyzed bisvinylogous 1,4-addition of cyclic 2,5-dienones to β-nitroolefins.

The remote ε-regioselective 1,4-addition of cyclic 2,5-dienones to nitroalkenes has been accomplished through in situ generation of chiral trienamine species derived from *Cinchona* amine **93**, combined to benzoic acid, leading to products **94** with moderate results (Scheme 22). Addition of tetramethylguanidine to the achieved products **94**, gave rise to an intramolecular Michael addition leading to the formation of chiral spirocyclic, or even bis-spirocyclic, compounds as single diastereomers [113].

**Scheme 23:**
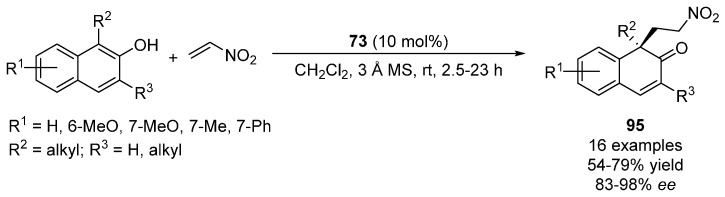
Asymmetric dearomatization of 2-naphthols through an organocatalyzed conjugate addition to nitroethylene.

Although not directly involving a ketone pro-nucleophiles, the catalytic asymmetric dearomatization (CADA) reaction of phenol derivatives is a procedure which leads to the generation of substituted cyclic enones [114]. Recently, 2-naphthols have been successfully used as nucleophiles in an organocatalytic conjugate addition to nitroethylene, affording enantioenriched functionalized 2-naphthalenones through a dearomatization process [115]. Thus, Takemoto’s thiourea organocatalyst 73 catalyzes this reaction affording the corresponding products 95 with high enantioselectivities (Scheme 23). A proposed highly ordered transition state, where a bifunctional hydrogen-bonding activation of the naphthol by the tertiary amine, and the electrophile by the thiourea, facilitates the *Re* face attack of the nucleophile.

### 2.3. 1,3-Dicarbonyl Compounds

**Scheme 24:**
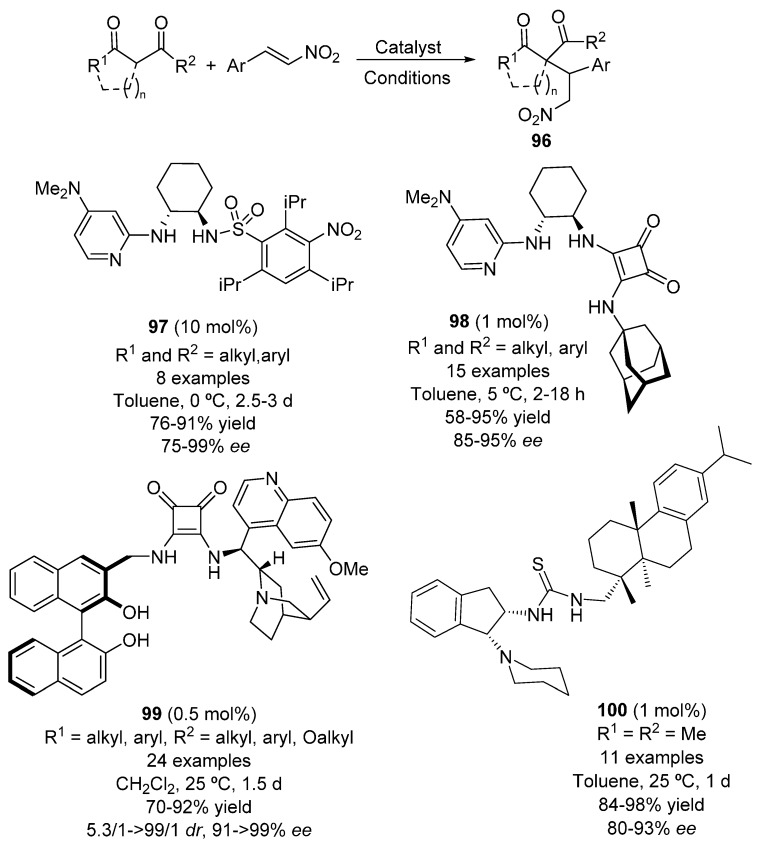
Asymmetric addition of 1,3-dicarbonyl compounds to nitroolefins using different organocatalysts.

The asymmetric addition of 1,3-dicarbonyl compounds onto nitroolefins has experienced an exponential growth since the beginning of this 21st century. The spectacular blossoming of research articles dealing with this transformation arises with the development of the asymmetric organocatalysis through hydrogen-bond activation. This fact somehow occurred after the publication in 2003 by Takemoto of a novel bifunctional thiourea-based organocatalyst (**73**) able to catalyze the enantioselective addition of malonates to nitroolefins with excellent yields and enantioselectivities [116]. Thus, the great performance of hydrogen-bonding organocatalysts in this transformation has prompted research groups worldwide for the development of new organic entities able to catalyze such process as well as for broadening the scope of dicarbonyl compounds employed. In the last years, different organocatalysts have been described to efficiently catalyze the addition of 1,3-diketones and β-keto esters to nitroalkenes, as it is chronologically illustrated in Scheme 24.

For example, catalyst **97** bearing sulfonamide/2-aminoDMAP moieties in its structure has proven to act as bifunctional acid/base organocatalyst in this reaction, obtaining the corresponding adducts in good yields and enantioselectivities [117]. Working in this idea, was published more recently the synthesis and evaluation of catalyst **98**, formed by the combination of squaramide and 2-aminoDMAP on a *trans*-cyclohexane-1,2-diamine as chiral scaffold [118]. The resulting catalyst was more active and able to reach similar yields and levels of enantioselection in the Michael products **96**, but using only 1 mol % loading. In addition, organocatalyst **99**, composed by BINOL, quinine and squaramide moieties, has turned out to be an extremely active catalyst for the enantioselective addition of a wide variety of 1,3-diketones and 1,3-keto esters onto nitroalkenes, since as low as 0.5 mol% catalyst loading was enough to promote such transformation in high yields, good to excellent diastereoselectivities and excellent *ee*’s [119]. Bifunctional organocatalysts **100**, which is based on rosin-derived indane amine has also demonstrated its high efficiency by promoting the addition of acetylacetone onto a wide variety of nitroalkenes (even aliphatic ones). Moreover, **100** is also able to catalyze the enantioselective addition of 2-hydroxy-1,4-naphthoquinone onto the same Michael acceptors [120].

**Figure 17:**
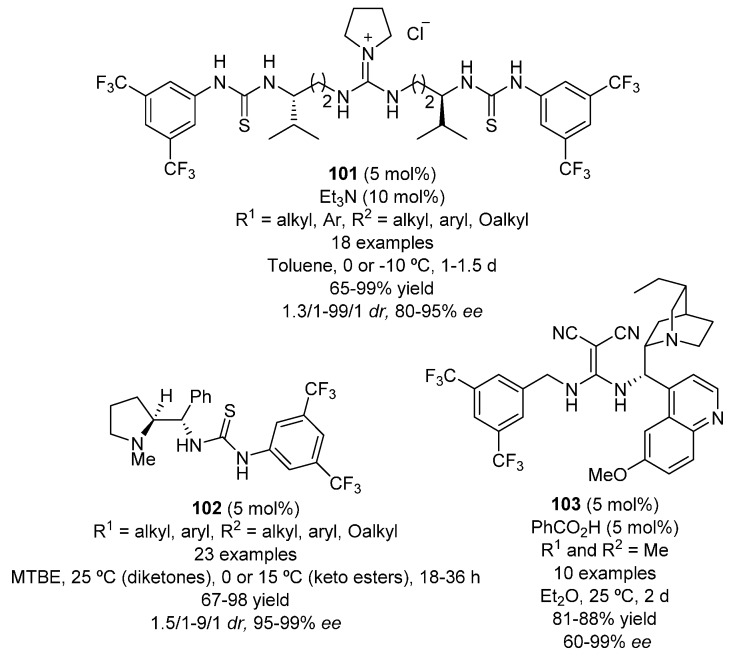
Organocatalyts employed in the asymmetric addition of 1,3-dicarbonyl compounds to nitroolefins leading to **96**.

1,3-Diamine-tethered guanidinium salt-thiourea **101** acted as a bifunctional catalyst able to efficiently catalyze this transformation leading to adduct **96** (Figure 17). In this case, the use of an external organic base like Et_3_N was necessary to accomplish good results [121]. In addition, the bifunctional thiourea-pyrrolidine catalyst **102**, synthesized from inexpensive l-proline in nine steps, also promotes addition of dicarbonyl compounds. Under optimized conditions, good to high yields and excellent enantioselectivities were achieved for adducts **96**. However, when non-symmetrically substituted dicarbonyl compounds were employed, the diastereoselectivities varied from poor to high [122]. Finally, organocatalyst **103**, based on a (diaminomethylene)malononitrile attached to a *Cinchona* motif has been reported for the asymmetric addition of acetylacetone to aryl nitroalkenes. Using such organocatalyst in combination with benzoic acid, moderate to high yields along with good enantioselectivities are obtained for the addition adducts **96** [123].

**Scheme 25:**
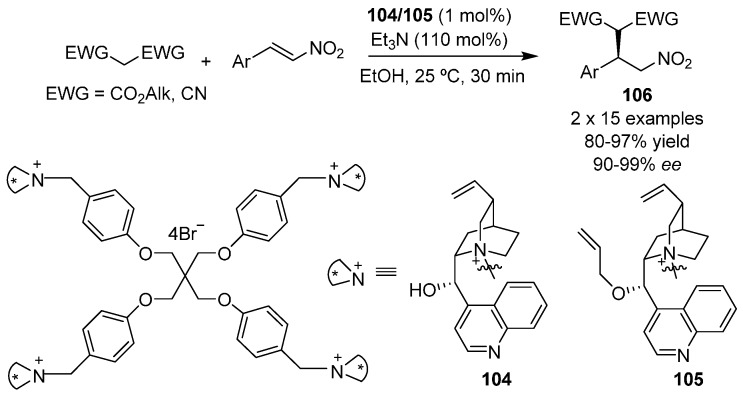
Asymmetric addition of malonates and malononitriles onto nitroolefins using organocatalysts **104** and **105**.

Organocatalysts **104** or **105** have been reported for the asymmetric addition of different malonates and malononitrile to aryl nitroalkenes. These catalysts, which are synthesized from pentaerythritrol-based tetrabromide, showed high effectiveness in only 1 mol% loading, affording excellent yields and enantioselectivities for adducts **106** (Scheme 25).

**Figure 18:**
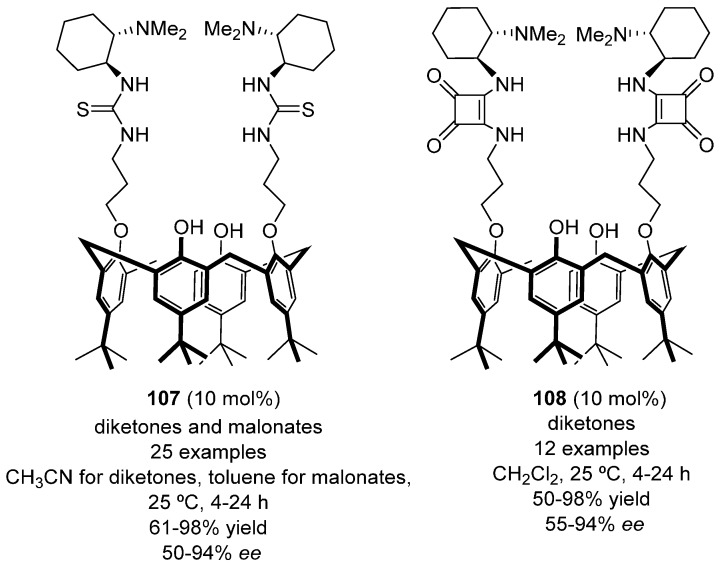
Calix[4]arene-based organocatalysts for the enantioselective addition of 1,3-diketones and malonates to nitroolefins.

The use of a stoichiometric amount of base was mandatory for reaching such results. This fact, along with the presence of quaternary ammonium salts, point to an ion-pair activation of the substrates, although H-bond interaction cannot be ruled out, especially with catalyst **104** bearing an OH group in its structure [124]. Two organocatalysts based on the use of calyx[4]arene as scaffold have recently been employed for this conjugate addition reaction (Figure 18). Thus, catalyst **107**, bearing a thiourea moiety was employed for the enantioselective addition of 1,3-diketones and malonates to nitroolefins, achieving moderate to high yields and enantioselectivities of the final adducts [125]. Similar results were obtained for the addition of 1,3-diketones when catalyst **108**, bearing a squaramide moiety in its structure was used [126].

**Scheme 26:**
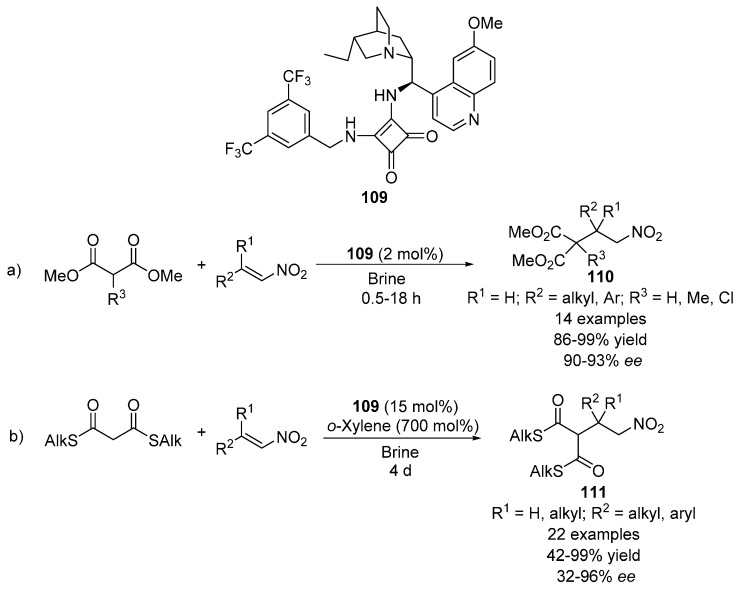
Asymmetric organocatalyzed addition of malonates and thiomalonates to nitroalkenes in brine.

As shown in previous examples, organocatalysts involving a hydrogen-bond activation normally require the use of non-polar aprotic solvents to obtain good results. Thus, hydrocarbons, ethers and chlorinated compounds are the main solvents employed. In this sense, an important breakthrough was the use of these organocatalysts in brine as environmentally benign reaction media. This highly polar solvent was not only tolerated for the Michael addition of dicarbonyl compounds onto nitroalkenes, but turned out to be beneficial for the reaction, since high yields and enantioselectivities of adducts **110** were obtained in short reaction times at room temperature using low catalyst loading of hydroquinine-squaramide derivative **109** (2 mol %) (Scheme 26a) [127]. The catalyst loading could be reduced to 0.01 mol% in some cases. The scope of this ‘on water’, protocol was successfully expanded to the addition of dithiomalonates to β,β-disubstituted nitroalkenes, allowing the one-pot synthesis of GABA-analogs bearing an all-carbon quaternary stereocenter starting from the resulting adducts **111**. In this latest case, a higher loading of **109** (15 mol %) was required along with the need for the addition of *o*-xylene as additive (Scheme 26b) [128]. This unexpected behavior was probably due to the so-called hydrophobic hydration effect. As a matter of fact, the same reaction carried out with the less hydrophobic quinine-squaramide analogue proceeded with lower yields and enantioselectivities.

**Scheme 27:**
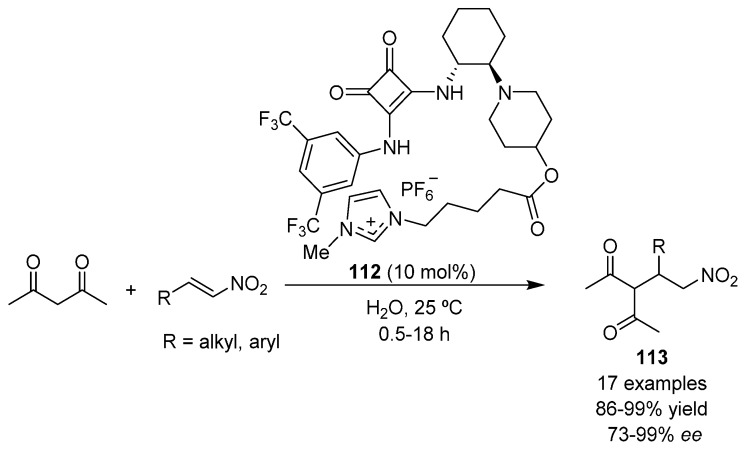
Enantioselective Michael addition ‘on water’ using recyclable organocatalyst **112**.

The use of a tertiary amine/squaramide derived organocatalyst **112** supported on an ionic liquid, allowed the use of pure water as solvent for the addition of acetylacetone to different nitroolefins due to its amphiphilic nature. The reaction proceeded in short reaction times with high yields and enantioselectivities (Scheme 27). The extraction of the Michael adducts **113** with an ether/*n*-hexane (8:2 *v*/*v*) mixture permitted the catalyst recycling over 30 times without a significant loss of activity [129].

**Scheme 28:**
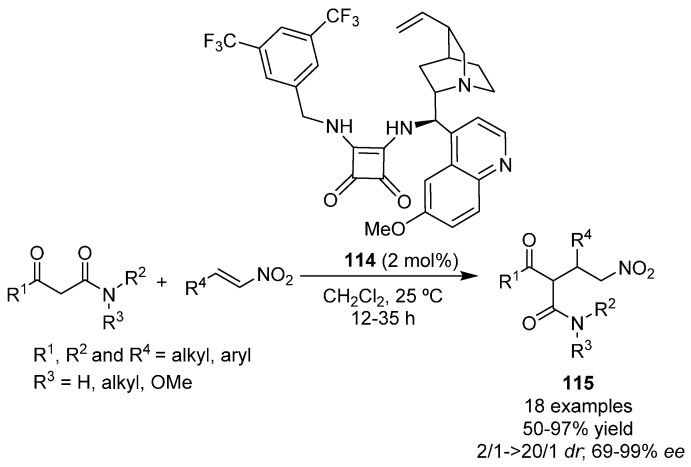
Organocatalyzed enantioselective addition of β-ketoamides to nitroalkenes.

As previously mentioned, one of the remaining challenges in the organocatalytic asymmetric addition of dicarbonyl compounds to nitroalkenes is the search for new pro-nucleophiles which allow further elaboration of the resulting adducts for the search of bioactive compounds. Thus, the quinine-squaramide derivative **114** has turned out to be an efficient organocatalyst for the enantio- and diastereoselective conjugate addition of β-ketoamides to aryl- and alkyl-nitroolefins, to afford adducts **115** (Scheme 28) [130]. These starting β-ketoamides, despite being more difficult to activate due to the lower acidity of the hydrogen placed in α-position, present some advantages compared to other dicarbonyl compounds. Thus, the lower acidity prevents a possible epimerization of the resulting Michael adducts. In addition, the presence of two substituents on the nitrogen allow to tune their steric and electronic properties more easily than other common 1,3-dicarbonyl pro-nucleophiles.

**Scheme 29:**
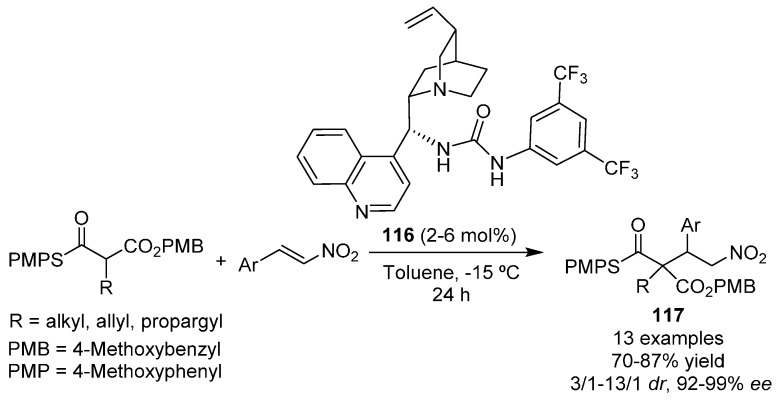
Organocatalyzed enantioselective addition of β-ketoamides.

The addition of *α*-substituted monothiomalonates to aryl nitroolefins have been recently described by using bifunctional *Cinchona* alkaloid-urea organocatalysts **116**. The corresponding adducts **117** were obtained with excellent *ee*’s along with diastereoselectivities ranging from moderate to good (Scheme 29) [131].

**Scheme 30:**
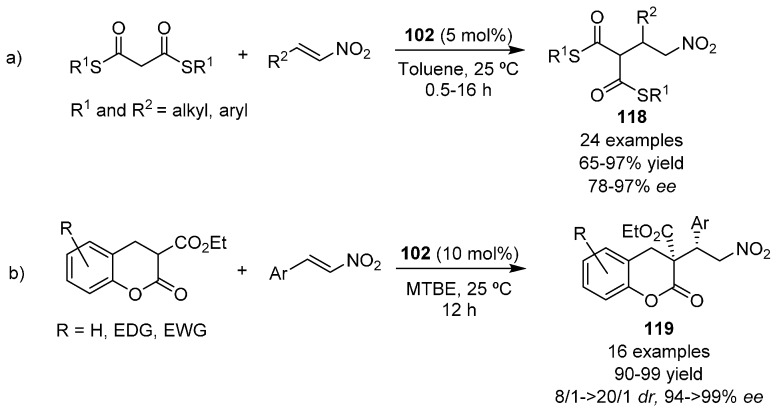
Enantioselective Michael addition of dithiomalonates and 2-oxochroman-3-carboxylate esters organocatalyzed by **102**.

The aforementioned thiourea-l-pyrrolidine derived tertiary amine **102**, has also demonstrated its effectiveness in the Michael addition of other dicarbonyl compounds, such as dithiomalonates [132] (Scheme 30a) and 2-oxochroman-3-carboxylate esters (Scheme 30b) [133] to nitroalkenes. The resulting adducts **118** and **119**, respectively, were obtained in excellent yields and enantioselectivities, and can be elaborated obtaining bioactive motifs. Organic molecules with a quaternary stereocenter bearing a fluorine atom are very attractive motifs for medicinal chemistry in the search of fluorinated analogs of bioactive compounds, their construction being challenging.

**Scheme 31:**
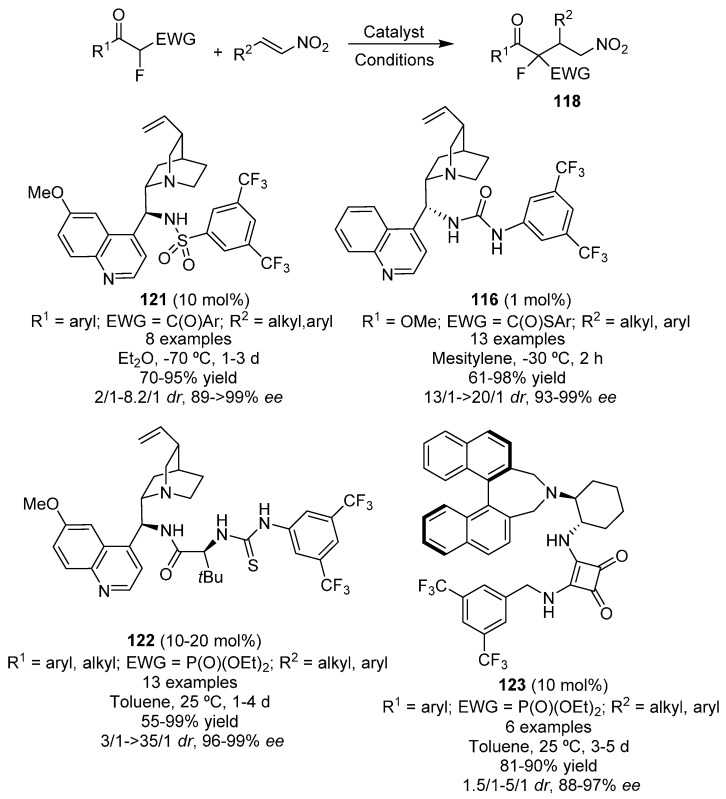
Organocatalyzed enantioselective Michael addition of fluorinated pro-nucleophiles to nitroolefins.

The use of unsymmetrically substituted 2-fluoro-1,3-dicarbonyl compounds as pronucleophiles in Michael additions to nitroolefins would render the formation of these enantioenriched compounds **120**, also bearing two consecutive all carbon stereocenters (Scheme 31).

Organocatalyst **121** has proven to be quite efficient in such purpose, allowing the reaction between 2-fluoro-1,3-diketones onto nitroalkenes in good yields and enantioselectivities but with moderate diastereoselectivities [134]. More recently, fluorinated monothiomalonates have been successfully added to nitroolefins in the presence of 1 mol % of organocatalyst **116**, obtaining excellent enantio- and diastereoselectivities for the corresponding products. Such thioesters are more labile than the corresponding esters and hence easier to be further transformed. In fact, after the reduction of the nitro group, the corresponding fluorinated lactams were synthesized in straightforward way [135].

Similarly, the use of α-fluoro β-ketophosphonates as active methylene compounds able to act as nucleophiles in this transformation has been also recently reported. Thus, both *Cinchona*-thiourea derivative **122** [136] and squaramide-based catalyst **123** [137] have proven to efficiently accomplish such purpose. Although good yields were observed using both catalysts, **122** turned out to be superior in terms of enantio- and diastereoselection (Scheme 31).

**Scheme 32:**
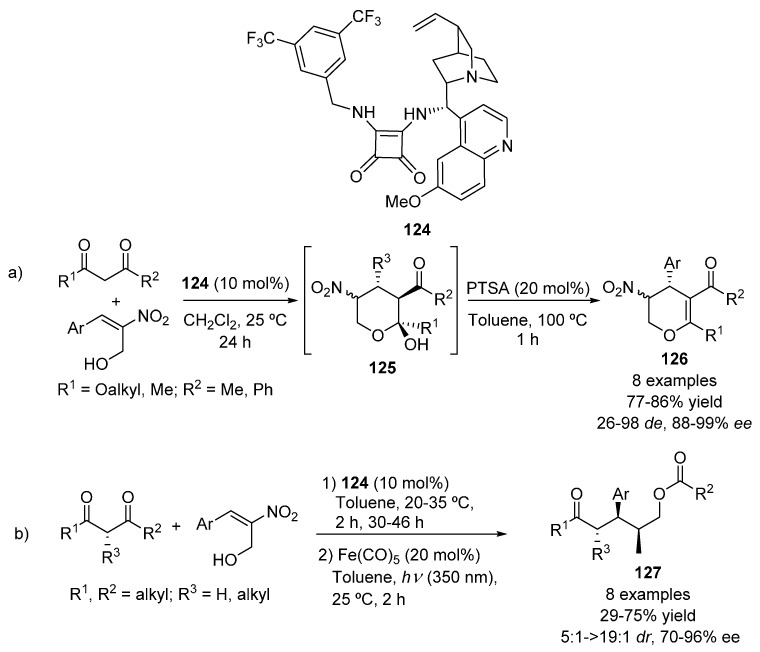
Organocatalyzed enantioselective Michael addition onto *α*-hydroxymethylnitroolefins.

Versatility and excellent performance as organocatalyst has shown *epi*-quinine-derived squaramide **124** in this kind of transformations, as illustrated in the examples depicted in Scheme 32. Thus, the synthesis of densely functionalized dihydropyrans **126** by dehydration of tetrahydropyrans **125**, which are obtained from the addition of 1,3-dicarbonyl compounds onto α-hydroxymethylated nitroolefins, is shown. The reaction proceeds via a domino Michael-hemiacetalization sequence, and the final heterocycles are obtained in high yields and enantioselectivities with a moderate to high diastereoselectivity (Scheme 32(a)) [138]. More recently, and based on the same idea, the synthesis of *O*-acyl-protected *γ*-hydroxy-nitroketones **127** has been described. Thus, the corresponding enantioenriched tetrahydropyrans **125** were submitted to an intramolecular Claisen-type fragmentation, catalyzed by photochemically activated Fe(0) complex. Products **127** were obtained in moderate yields with high enantio- and diastereoselectivities (Scheme 32b) [139].

**Scheme 33:**
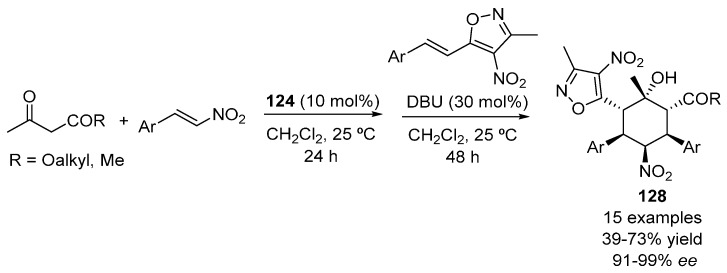
Multicomponent reaction based on the organocatalyzed enantioselective Michael addition of 1,3-dicarbonyl compounds to nitroolefins catalyzed by **124**.

Finally, it is important to remark that the same catalyst **124** has been used for the synthesis of complex molecules bearing up to six consecutive stereocenters in a one-pot multicomponent reaction. Thus, highly functionalized cyclohexane-derivatives **128** can be obtained starting from an asymmetric organocatalyzed 1,4-addition of 1,3-dicarbonyl to nitroolefins, followed by a 1,6-/1,2 addition sequence in the presence of an achiral organic-base. Despite the complexity of the products, moderate to good yields were obtained along with excellent enantioselectivities in products **128**, using only 1 mol % of organocatalyst (Scheme 33) [140].

### 2.4. Nitroalkanes

**Scheme 34:**
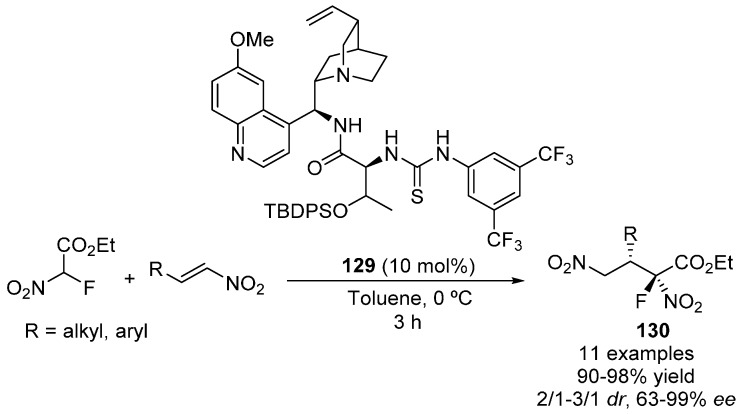
Asymmetric Michael addition of α-fluoro-α-nitro esters to nitroalkenes catalyzed by an alkaloid-threonine-thiourea derivative.

The asymmetric Michael addition of α-fluoro-α-nitro esters to nitroolefins for the preparation of compounds **130** has recently been reported employing a *Cinchona*-alkaloid-amino acid-thiourea structure **129** as organocatalyst (Scheme 34) [141]. Different conditions for the nitro reduction of compounds **130** were assayed to obtain the corresponding α-fluoro-α-amino acid derivatives, but only the use of H_2_ in the presence of Pd/BaSO_4_ gave partial success (20% yield). Denitration of one of the adducts **130** was accomplished using radical conditions.

**Scheme 35:**
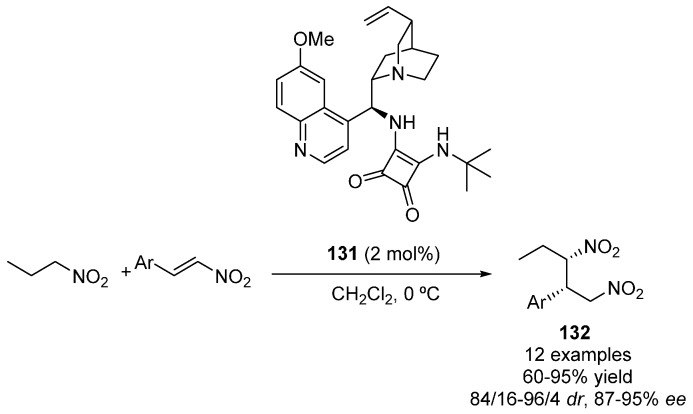
Asymmetric Michael addition of 1-nitropropane to nitroalkenes organocatalyzed by an alkaloid-squaramide derivative.

A chiral bifunctional alkaloid-squaramide organocatalyst **131** has been used for the Michael addition of 1-nitropropane to different nitroalkenes [142]. The conjugate addition products **132** were obtained in high to excellent yields and stereoselectivities, being insensitive to the electronic nature of the aromatic ring (Scheme 35).

### 2.5. Heterocyclic Compounds

**Scheme 36:**
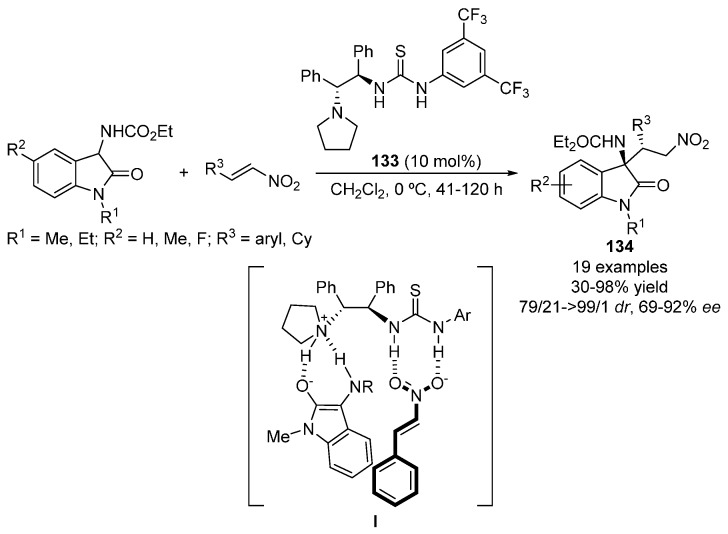
Asymmetric organocatalytic conjugate addition of 3-aminooxindoles to nitroolefins.

Chiral oxindoles have attracted significant attention in asymmetric catalysis and medicinal chemistry due to their prevalence in a variety of natural and biologically active compounds [143,144]. Particularly, chiral 3,3-disubstituted oxindole derivatives are considered crucial scaffolds in several drugs [145,146]. Thus, 3-functionalized oxindoles have been efficiently used as nucleophiles in the conjugate addition to nitrostyrene derivatives. As usual, the reaction is organocatalyzed by chiral bifunctional organocatalysts involving a dual Brønsted base/hydrogen bonding activation of the nucleophile and the electrophile. An example is the thiourea-tertiary amine catalyst **133** which promotes the conjugate addition of 3-aminooxindoles to nitroolefins, affording the corresponding derivatives **134** bearing a quaternary stereogenic center with high yields, diastereo- and enantioselectivities (Scheme 36) [147]. Interestingly, high selectivities (>99/1 *dr*, 87% *ee*) were also obtained for aliphatic nitroolefins such as (*E*)-(2-nitrovinyl)cyclohexane (R^3^ = cyclohexyl), although in low yields (30%). A dual and simultaneous Brønsted base/hydrogen bonding activation of the nucleophile and electrophile, respectively, has been proposed based on related activation models and the observed product stereochemistry, as depicted in the model **I** (Scheme 36).

**Scheme 37:**
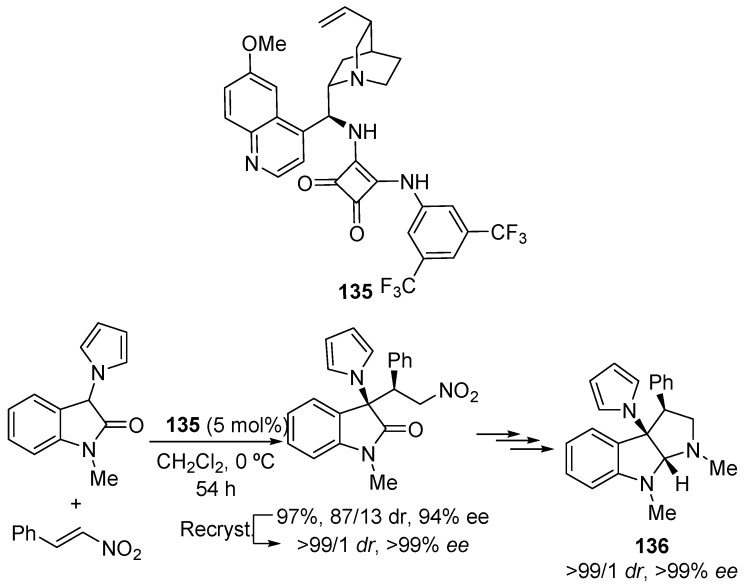
Asymmetric organocatalytic conjugate addition of 3-aminooxindoles to nitroolefins.

A range of chiral 3-pyrrolyl-3,3’-disubstituted oxindoles have been prepared via conjugate addition of 3-pyrrolyloxindoles to β-nitrostyrene organocatalyzed by the *Cinchona*-alkaloid squaramide-based organocatalyst **135** [148]. The reaction affords the corresponding products in high yields, moderate diastereoselectivities, and good to excellent enantioselectivities for the major diastereoisomer. The usefulness of the methodology has been demonstrated by the synthesis of different optically active compounds, such as the pyrrolidinoindoline derivative **136**, with a core structure related to interesting natural products, such as (−)-physostigmine and (−)-pseudophrynaminol (Scheme 37).

**Figure 19:**
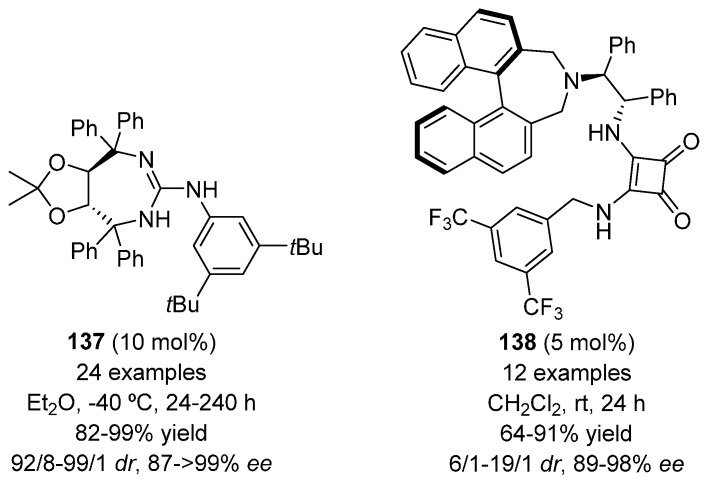
Organocatalysts employed for the conjugate addition reactions of 3-alkyloxindoles and 3-chlorooxindoles to nitroolefins.

The asymmetric Michael addition reaction of 3-alkyloxindoles [149] or 3-chlorooxindoles [150] to nitroalkenes has been explored recently using as organocatalysts the guanidine **137** and the squaramide **138**, respectively. The reaction conditions, yields and stereoselectivities of the corresponding adducts are shown in Figure 19.

**Scheme 38:**
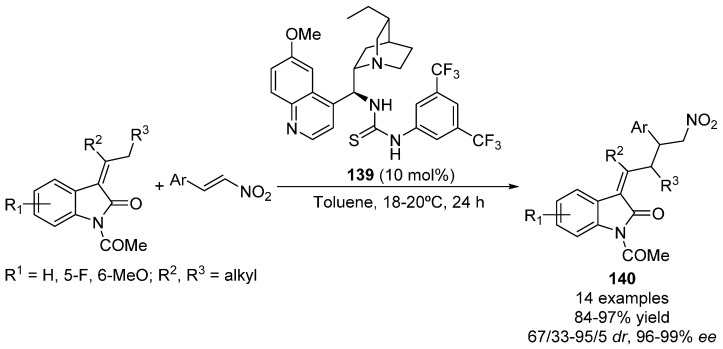
Asymmetric organocatalyzed conjugate addition of 3-alkylideneoxindoles to nitroolefins.

3-Alkylideneoxindoles have also been studied in the conjugate addition to nitroolefins [151]. The ability of 3-alkylideneoxindoles to react as electron-poor acceptors converts these substrates in challenging nucleophiles. Thus, dihydroquinine-derived thiourea organocatalyst **139** promotes the asymmetric vinylogous Michael addition of a variety of 3-alkylideneoxindoles to β-nitrostyrene derivatives through a bifunctional activation mechanism, to afford the corresponding products **140** with excellent regio- and stereoselectivities, no olefin isomerization being observed (Scheme 38). In addition, thiourea **139** also organocatalyzed the conjugate addition of α-alkylidenepyrazolidinones to nitroolefins [152].

**Scheme 39:**
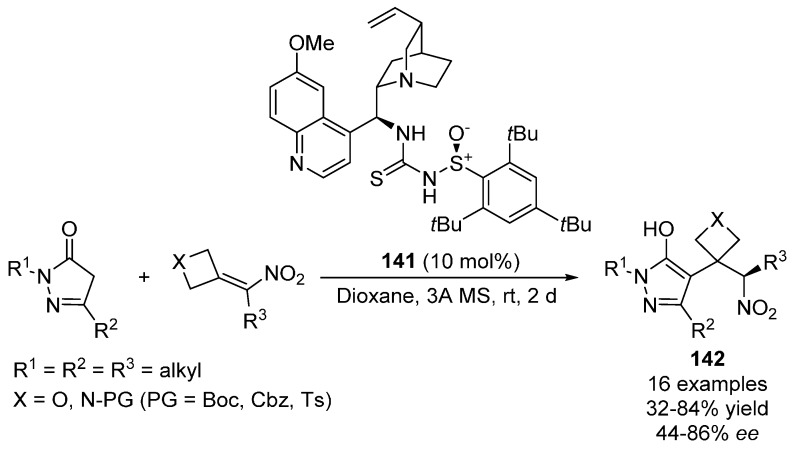
Asymmetric organocatalyzed conjugate addition of pyrazolones to four-member heterocycle-containing nitroolefins.

Due to the frequent occurrence of the pyrazolone core in many important naturally occurring compounds and drug molecules, great efforts have been made for their chiral synthesis [153]. So far, numerous works have been reported on the pyrazolone addition, taking advantage of the aromatic character acquired by these substrates after deprotonation. Interestingly, challenging nitroolefins have been used as electrophiles for the conjugate addition. For instance, the *N*-sulfinylurea organocatalyst **141** has been employed to promote the addition of different 3-substituted pyrazolone derivatives to nitroalkenes bearing an oxetane or azetidine ring at the β-position (Scheme 39) [154]. The nitroalkane pyrazolone adducts **142** are obtained with moderate to good yields and enantioselectivities.

**Scheme 40:**
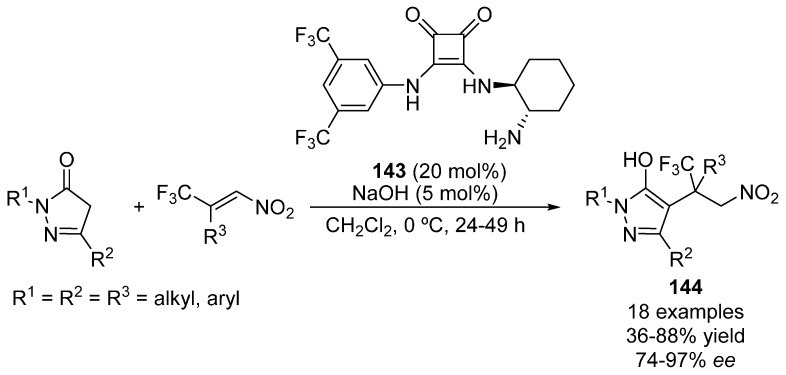
Organocatalyzed asymmetric conjugate addition of pyrazolones to trifluoromethylated nitroolefins.

Chiral squaramide **143** has been successfully used as organocatalyst in the conjugate addition of 3-substituted pyrazolones to β-trifluoromethyl β-disubstituted nitroolefins to afford the corresponding Michael adducts **144** in moderate to good yields and good to excellent enantioselectivities [155] (Scheme 40). However, the reaction is quite unpredictable in terms of electronic effects. For instance, β-nitrostyrenes with both strong electron-withdrawing (-CF_3_) or donating (-OMe) groups at the para position of the phenyl ring do not show any reactivity in the process. Also, striking effects on the reaction yield and enantioselectivity have been observed depending on the electronic features of the substituents of the phenyl ring at the 1-position of the pyrazolinone. Thus, halogenated (4-ClC_6_H_4_, 4-BrC_6_H_4_) as well as electron-donating (4-MeOC_6_H_4_) *para* substituents afforded the addition products in moderate to good yields and excellent enantioselectivities, while strong electron-withdrawing groups (-CF_3_) led to low yields. A bifunctional activation of the deprotonated tautomer of the nucleophile and the electrophile by the chiral catalyst is proposed in the reaction transition state.

**Scheme 41:**
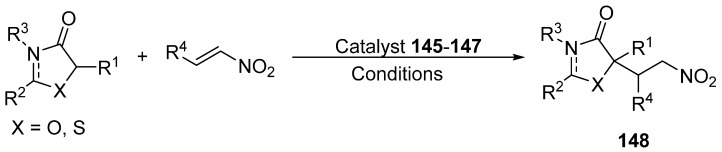
Asymmetric organocatalyzed conjugate addition of oxazolones, thiazolones, oxazolidindiones, and thiazolidindiones to nitroolefins.

**Figure 20:**
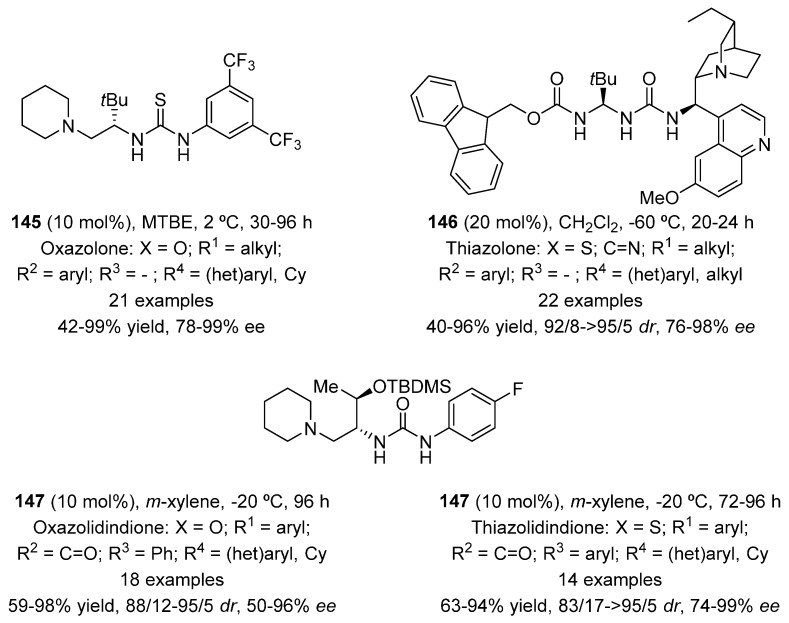
Organocatalysts employed in the conjugate addition of oxazolones, thiazolones, oxazolidindiones, and thiazolidindiones to nitroolefins leading to **148**.

Chiral urea- and thiourea-based organocatalysts **145–147** have been reported to organocatalyze the conjugate addition of oxazolones [156], thiazolones [157], oxazolidindiones [158], and thiazolidindiones [159] to nitroalkenes leading to the corresponding adducts 148 (Scheme 41, Figure 20). In general, high yields and selectivities are observed for all the tested nucleophiles and β-arylated nitroalkenes, whereas the use of aliphatic nitroolefins usually affords low yields irrespective of the employed nucleophile (Figure 20).

**Scheme 42:**
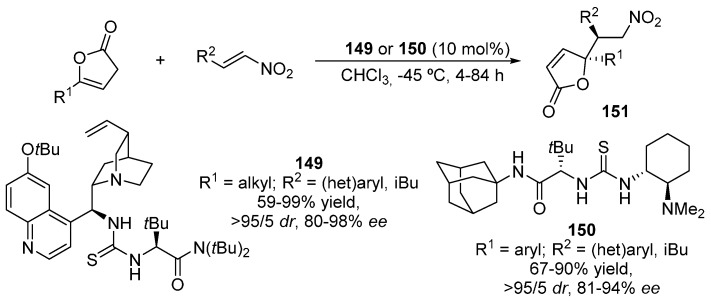
Asymmetric organocatalyzed direct vinylogous conjugate addition of deconjugated butenolides to nitroolefins.

Lactones [160,161], azlactones [162], phthalides [163], cyclic hemiacetals [164], and benzofuranones [165] have shown as suitable pro-nucleophiles in the asymmetric organocatalytic conjugate addition to nitroolefins. Concerning lactones, the direct asymmetric vinylogous conjugate addition of γ-aryl-substituted deconjugated butenolides to β-alkyl and β-aryl substituted nitroolefins has been performed using chiral thioureas **149** and **150** as organocatalysts, to afford the corresponding chiral lactones **151** bearing adjacent quaternary and tertiary stereocenters with good isolated yields, high diastereoselectivities, and excellent enantioselectivities [160] (Scheme 42).

**Scheme 43:**
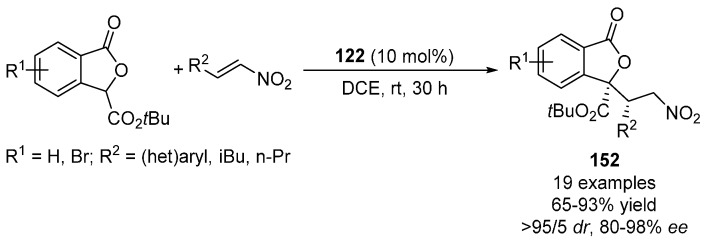
Asymmetric organocatalyzed Conjugate addition of phthalide derivatives to nitroolefins.

Chiral phthalides are present in numerous natural products and drugs [166]. An interesting asymmetric organocatalyzed conjugate addition of phthalide derivatives to nitroolefins has been recently reported [163]. The reaction, catalyzed by quinine-derived thiourea **122**, is carried out in dichloroethane (DCE) at room temperature, the corresponding functionalized 3,3-disubstituted phthalide derivatives **152** being obtained in high yields, diastereo- and enantioselectivities (Scheme 43).

**Scheme 44:**
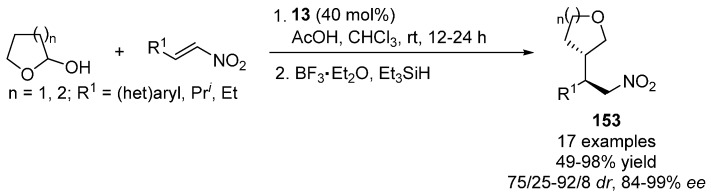
Organocatalytic enantioselective addition of cyclic hemiacetals to nitroolefins.

A highly enantioselective Michael addition of cyclic hemiacetals to nitroolefins has recently been reported using the Hayashi-Jørgensen proline-derived catalyst 13 [164]. This methodology allows the preparation of optically active 3-substituted tetrahydrofurans and tetrahydropyrans 153, which are compounds not as easily accessible as the 2-substituted derivatives. This catalytic methodology is of high synthetic utility, allowing the synthesis of 2,3-disubstituted cyclic ethers, α-substituted lactones, and analogues of the antidepressant venlafaxine (Scheme 44).

## 3. Nitrogen Nucleophiles

**Scheme 45:**
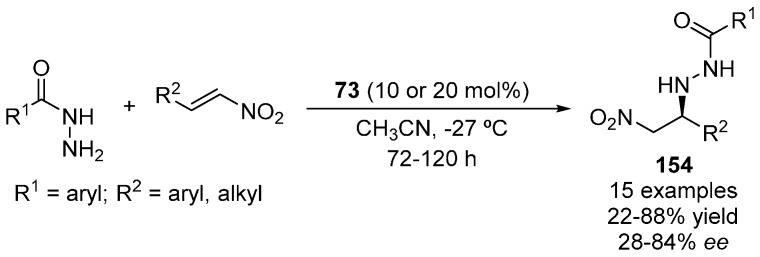
Asymmetric aza-Michael addition of acylhydrazines to nitroalkenes by using Takemoto’s thiourea catalyst **73**.

The organocatalytic aza-Michael reaction has attracted considerable attention in recent years [167,168,169,170,171]. Among the employed nucleophiles, the most frequently studied have been carbamates, benzotriazoles, pyrazolinones, hydrazones and hydroxylamines. More recently, the use of organocatalysts having a tertiary amine and a thiourea moiety has allowed the Michael addition of nitrogen nucleophiles, a double activation role being assigned to these catalysts. Thus, Takemoto’s organocatalyst **73** has been employed in the addition of acylhydrazines to nitroalkenes giving the products **154** (Scheme 45). Both electron-rich and electron-poor aryl nitroalkenes were successfully employed obtaining good enantioselectivities, whereas rather poor results were obtained using aliphatic nitroalkenes [172].

**Scheme 46:**
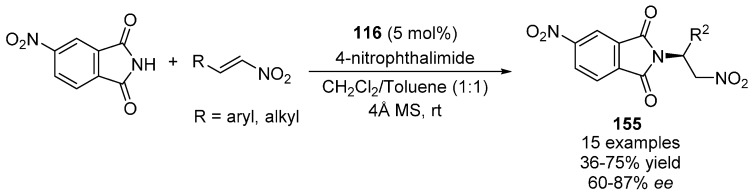
Asymmetric aza-Michael addition of 4-nitrophthalimide to nitroalkenes using an alkaloid-thiourea derivative as organocatalyst.

Nitrogen containing heterocycles, such as phthalimide and benzotriazole have been added successfully to nitroolefins mediated by a *Cinchona*-alkaloid thiourea organocatalyst. Thus, 4-nitrophthalimide reacted with a variety of nitroalkenes in the presence of the alkaloid-thiourea **116** to give the corresponding Michael adducts **155** (Scheme 46) [173]. Activity test showed that some of these products have moderate or good herbicidal activity against cole and barnyard grass.

**Scheme 47:**
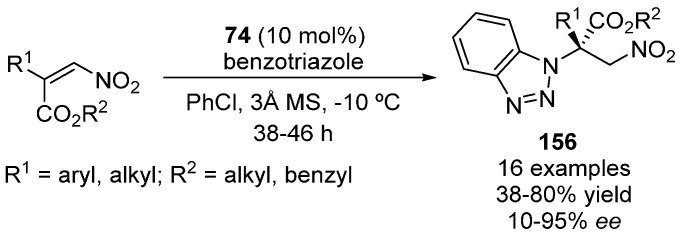
Asymmetric aza-Michael addition of benzotriazole to β,β-disubstituted nitroalkenes by using an alkaloid-thiourea derivative.

The formation of quaternary stereogenic centers has been achieved using organocatalyst **74** via an aza-Michael addition of benzotriazole to the alkoxycarbonylated nitroalkenes [174], the corresponding adducts **156** being obtained in moderate to high yields and with high enantioselectivities (Scheme 47).

**Scheme 48:**
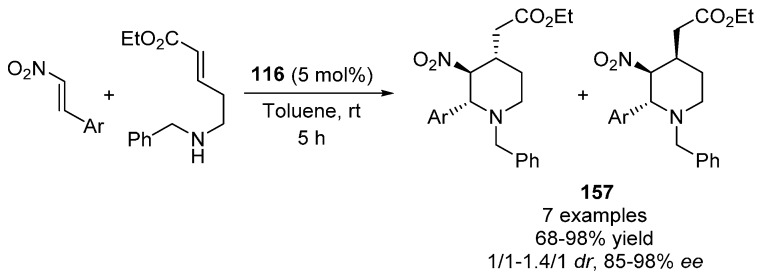
Asymmetric cascade aza-Michael/Michael addition for the synthesis of piperidines by using an alkaloid-thiourea derivative.

5-Aminopent-2-enoate derivatives have been employed as nitrogen nucleophiles in the aza-Michael addition to nitrostyrenes. The formed adduct underwent a second Michael addition mediated by the same catalyst to give tetrasubstituted piperidines **157** [175]. Different *Cinchona*-alkaloid-based thioureas were tested as organocatalysts in this transformation, giving similar results: low diastereoselectivity and high enantioselectivity. Catalyst **116** proved to be slightly superior to the others in the asymmetric cascade aza-Michael/Michael addition using nitroalkenes (Scheme 48). The obtained diastereoisomers **157** were inseparable, which represents a drawback for this transformation.

## 4. Oxygen Nucleophiles

**Scheme 49:**
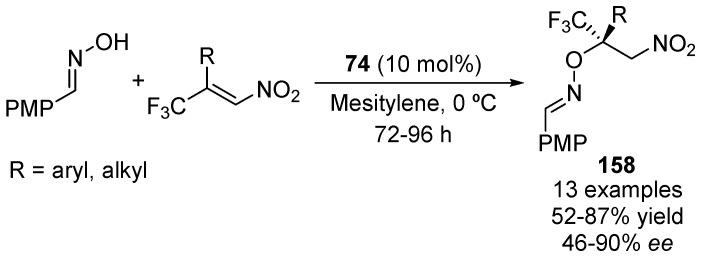
Asymmetric oxa-Michael addition of 4-methoxybenzaldehyde oxime to nitroalkenes by using an alkaloid-thiourea derivative.

The asymmetric conjugated addition of oxa-nucleophiles has been described by means of alkaloid based thiourea organocatalysts, being a very straightforward approach in the formation of carbon-oxygen bonds [171]. Oximes have proved to be suitable oxygen-centered nucleophiles in the Michael addition to nitroalkenes bearing a trifluoromethyl substituent [176]. Among the organocatalysts tested, **74** gave the best yields and stereoselectivities in the addition of 4-methoxybenzaldehyde oxime to a variety of β,β-disubstituted nitroalkenes, affording adducts **158** (Scheme 49). Analogous catalysts bearing urea or squaramide moieties instead of the thiourea unit resulted less productive in terms of conversion and selectivity.

**Scheme 50:**
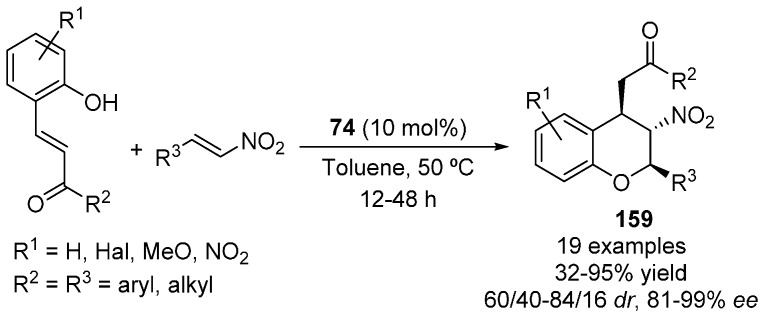
Asymmetric cascade oxa-Michael/Michael addition by using an alkaloid-thiourea derivative as organocatalyst.

The same *Cinchona*-derived thiourea organocatalyst **74** has been employed in the preparation of chromans by means of an oxa-Michael/Michael cascade reaction [177]. Thus, aryl 2-(2-hydroxy- phenyl)vinyl ketones reacted with nitroalkenes in an apolar solvent, such as toluene, favoring the activation of the substrates by hydrogen-bonding. The adducts **159** were obtained with high selectivities independently of the substituents in the nitrostyrenes, although electron-withdrawing groups enhanced the reactivity, yielding the expected products in shorter reaction times (Scheme 50). On the contrary, the substituents on the ketone did not show any effect in the outcome of the reaction. This asymmetric synthesis of chromans has also been performed using β-CF_3_-nitroalkenes as electrophiles, using a *Cinchona*-derived squaramide as organocatalyst [178].

## 5. Sulfur Nucleophiles

**Scheme 51:**
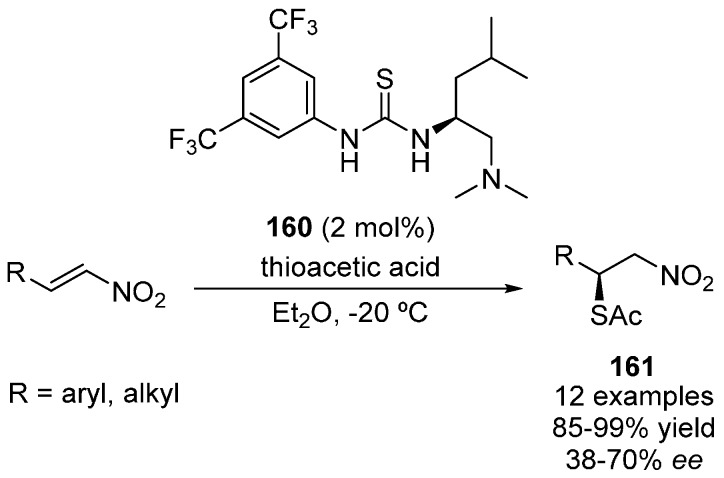
Asymmetric sulfa-Michael addition of thioacetic acid to nitroalkenes by using a tertiary amine-thiourea derivative as organocatalyst.

**Figure 21:**
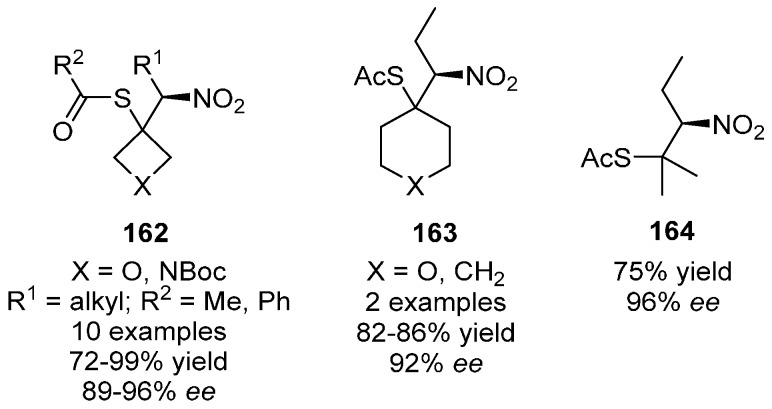
Enantioenriched 1,2-nitrothioesters via addition of thioacids to nitroalkenes organocatalyzed by **74**.

The asymmetric sulfa-Michael addition to activated olefins allows the preparation of S-centered optically active compounds, thioacetic acid being commonly employed as sulfur nucleophile [171]. Thus, thioacetic acid has been added to nitroalkenes using the amine-thiourea organocatalyst **160**, yielding the corresponding adducts **161** with moderate enantioselectivities (Scheme 51) [179]. In addition, the alkaloid-thiourea **74** has also shown to be active and selective in the addition of thioacids to trisubstituted nitroalkenes [180]. Thus, oxetane and azetidine nitroalkenes reacted with thioacetic and thiobenzoic acids at low temperature (−78 °C), giving the corresponding 1,2-nitrothioesters **162**–**164** with high yield and enantioselectivities (Figure 21). The nitroalkene bearing a 4-membered ring gave rise to the higher reactivity, whereas the reaction with substrates bearing 6-membered ring or acyclic moieties needed a higher temperature (−25 °C) to give the corresponding addition products.

**Scheme 52:**
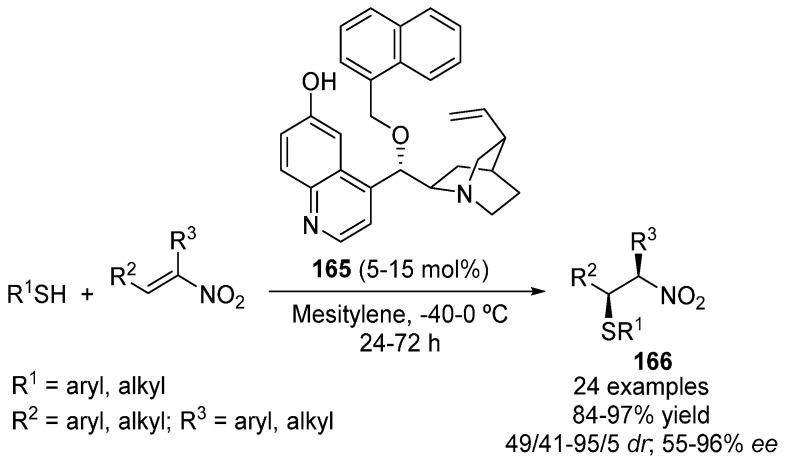
Asymmetric sulfa-Michael addition of thiols to nitroalkenes by using an alkaloid derivative.

Different aryl and alkyl thiols have been added to 1,2-disubstituted nitroalkenes using the modified *Cinchona* alkaloid **165** in a nonpolar solvent. Sulfa-Michael adducts **166** were obtained in excellent yields with good diastereoselectivities and high enantioselectivities, independently of the substitution pattern on the nitro compound or the S-nucleophile (Scheme 52) [181].

**Scheme 53:**
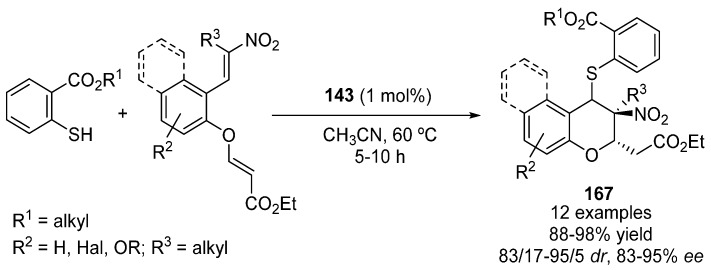
Asymmetric cascade sulfa-Michael/Michael addition leading to chromans by using a cyclohexanediamine-squaramide organocatalyst.

The enantioselective synthesis of chroman and thiochroman derivatives has been achieved by an asymmetric cascade sulfa-Michael/Michael addition starting from different thiophenol derivatives, squaramide-based organocatalysts being employed in both transformations. Thus, the synthesis of chromans **167** has been achieved using organocatalyst **143** (Scheme 53), although other tertiary amine-squaramide catalysts also showed good activity [182].

**Scheme 54:**
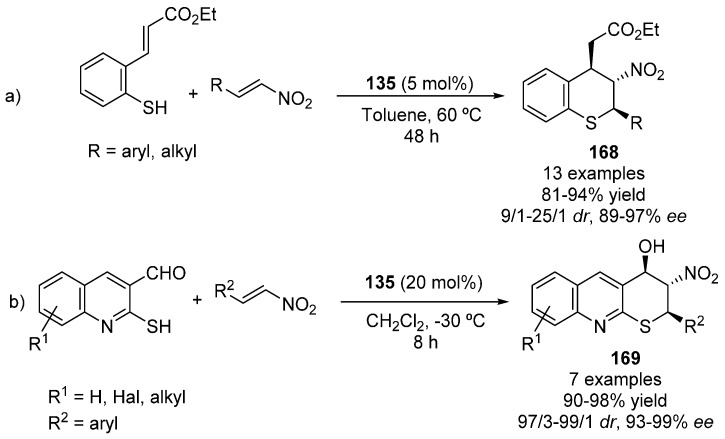
Asymmetric cascade sulfa-Michael/Michael addition leading to thiochromans by using an alkaloid-thiourea derivative as organocatalyst.

Among the squaramide-based organocatalysts, the alkaloid-squaramide catalyst **135** has provided the best results in the preparation of thiochromans **168** (Scheme 54a) [183]. Following the same idea and type of catalyst, the sulfa-Michael addition to a nitroalkene can be followed by a Henry reaction, allowing the preparation of thiopyran motifs in an enantioselective manner as described in the synthesis of dihydrothiopyrano[2,3-*b*]quinolines **169** (Scheme 54b) [184]. For the latter case, the use of another alkaloid-based organocatalysts has allowed to reverse the diastereoselectivity of the transformation.

## 6. Phosphorous Nucleophiles

**Scheme 55:**
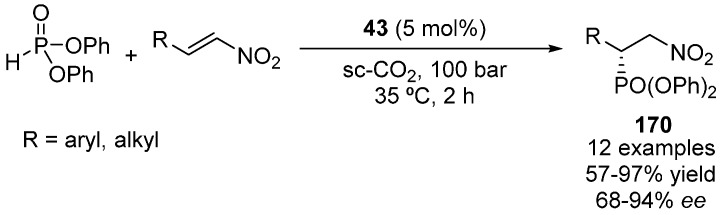
Asymmetric phospha-Michael addition of diphenylphosphite to nitroalkenes by using a tertiary amine-squaramide derivative as organocatalyst.

The phospha-Michael addition to nitroalkenes has been recently described using diphenylphosphite as P-centered nucleophile [185]. Thus, a tertiary amine-squaramide and a tertiary amine-thiourea have been assayed as bifunctional organocatalysts in this asymmetric reaction in a supercritical carbon dioxide medium. The squaramide unit exhibited better activity and selectivity in this transformation, the best catalytic performance being obtained when using squaramide **43** as organocatalyst. Following this procedure, nitrophosphonates **170** have been obtained in high yields and enantioselectivities (Scheme 55).

## 7. Conclusions

Asymmetric organocatalysis is a very powerful synthetic tool that, when applied to the conjugate addition of nucleophiles to nitroalkenes can prepare valuable final products in high yields and stereoselectivities, usually employing a simple methodology. Those final adducts bear a nitro functionality, which is amenable of further synthetic transformations towards interesting compounds. This revision has shown how active is nowadays this field. A huge array of organocatalysts have been developed recently to perform this conjugate addition reaction employing many different nucleophiles and achieving impressive results in many cases. However, many challenges persist and still improvements are necessary. Thus, usually the higher enantioinductions are achieved when using β-arylated nitroolefins as electrophiles, their alkylated counterparts frequently being less effective. In addition, polysubstituted nitroalkenes have been less explored. Moreover, although numerous organocatalysts have been developed, many times they are only employed in model reactions. Additional research concerning the development of organocatalysts able to promote high stereoselectivities using different nucleophiles and nitroolefins, low loadings and environmentally friendly conditions is necessary. Recoverable and reusable catalytic systems would add an additional value to the methodology, increasing the possibility of industrial applications. With all these considerations, no doubt that asymmetric organocatalytic conjugate addition to nitroolefins will continue to be a fast-forwarding topic in the next future.
